# Cul-4 inhibition rescues spastin levels and reduces defects in hereditary spastic paraplegia models

**DOI:** 10.1093/brain/awae095

**Published:** 2024-03-29

**Authors:** Francesca Sardina, Claudia Carsetti, Ludovica Giorgini, Gaia Fattorini, Gianluca Cestra, Cinzia Rinaldo

**Affiliations:** National Research Council (CNR), Institute of Molecular Biology and Pathology (IBPM), c/o University of Rome Sapienza, 00185 Rome, Italy; National Research Council (CNR), Institute of Molecular Biology and Pathology (IBPM), c/o University of Rome Sapienza, 00185 Rome, Italy; Department of Biology and Biotechnology, University of Rome Sapienza, 00185, Rome, Italy; National Research Council (CNR), Institute of Molecular Biology and Pathology (IBPM), c/o University of Rome Sapienza, 00185 Rome, Italy; National Research Council (CNR), Institute of Molecular Biology and Pathology (IBPM), c/o University of Rome Sapienza, 00185 Rome, Italy; Department of Biology and Biotechnology, University of Rome Sapienza, 00185, Rome, Italy; National Research Council (CNR), Institute of Molecular Biology and Pathology (IBPM), c/o University of Rome Sapienza, 00185 Rome, Italy; Department of Biology and Biotechnology, University of Rome Sapienza, 00185, Rome, Italy; Fondazione Santa Lucia IRCCS, c/o CERC, 00179, Rome, Italy; National Research Council (CNR), Institute of Molecular Biology and Pathology (IBPM), c/o University of Rome Sapienza, 00185 Rome, Italy

**Keywords:** spastin, hereditary spastic paraplegia, poly-ubiquitination, CRL4, Drosophila neurodegenerative model

## Abstract

Hereditary spastic paraplegias (HSPs) are degenerative motor neuron diseases characterized by progressive spasticity and weakness in the lower limbs. The most common form of HSP is due to SPG4 gene haploinsufficiency. SPG4 encodes the microtubule severing enzyme spastin. Although, there is no cure for SPG4-HSP, strategies to induce a spastin recovery are emerging as promising therapeutic approaches. Spastin protein levels are regulated by poly-ubiquitination and proteasomal-mediated degradation, in a neddylation-dependent manner. However, the molecular players involved in this regulation are unknown.

Here, we show that the Cullin-4-RING E3 ubiquitin ligase complex (CRL4) regulates spastin stability. Inhibition of CRL4 increases spastin levels by preventing its poly-ubiquitination and subsequent degradation in spastin-proficient and in patient derived SPG4 haploinsufficient cells. To evaluate the role of CRL4 complex in spastin regulation *in vivo*, we developed a *Drosophila melanogaster* model of SPG4 haploinsufficiency which show alterations of synapse morphology and locomotor activity, recapitulating phenotypical defects observed in patients. Downregulation of the CRL4 complex, highly conserved in *Drosophila*, rescues spastin levels and the phenotypical defects observed in flies. As a proof of concept of possible pharmacological treatments, we demonstrate a recovery of spastin levels and amelioration of the SPG4-HSP-associated defects both in the fly model and in patient-derived cells by chemical inactivation of the CRL4 complex with NSC1892.

Taken together, these findings show that CRL4 contributes to spastin stability regulation and that it is possible to induce spastin recovery and rescue of SPG4-HSP defects by blocking the CRL4-mediated spastin degradation.

## Teaser

Restoring spastin protein levels could be beneficial for patients with hereditary spastic paraplegia. Sardina *et al.* identify a new druggable pathway regulating spastin stability, and show in preclinical models that inhibiting this pathway prevents spastin degradation, leading to higher spastin levels and reduced phenotypic defects.

## Introduction

Hereditary spastic paraplegias (HSPs) are neurodegenerative diseases characterized by progressive spasticity of the lower extremities due to axonal degeneration in the corticospinal motor tracts.^1,2^ Spastic paraplegia type 4 [SPG4, Online Mendelian Inheritance in Man (OMIM) #182601] is the most frequent form, accounting for 40%–60% of autosomal dominant and 20% of sporadic HSPs. SPG4-HSP is caused by heterozygous loss-of-function mutations in the SPG4 gene, encoding spastin.^3-5^ Spastin is a microtubule (MT)-severing enzyme that regulates MT remodelling and dynamics^5-7^ and controls cytokinesis and membrane trafficking, with relevant effects on the axonal transport.^6-9^ More than 250 mutations of the SPG4 gene have been reported, which include missense (clustering mainly in the catalytic domain) and truncating (frameshift, nonsense and insertions/deletions) mutations, which are commonly associated with reduced spastin protein levels as a result of mRNA nonsense-mediated decay. Therefore, SPG4-HSP is thought to be mostly due to spastin haploinsufficiency and reduced MT severing activity^10-13^; other mechanisms, such as dominant negative effects and gain-of-toxic function, appear to be potentially relevant only for a subpopulation of patients.^14,15^ Human spastin has two main isoforms, M1 and M87, encoded by different translation start sites on the same mRNA, and both isoforms might undergo exon 4 skipping.^16^ M87 is the most abundant ubiquitous variant, while M1, although present in many tissues, is enriched mainly in neuronal tissues. The presence of different spastin protein variants has been reported in vertebrates and invertebrates and is conserved in *Drosophila melanogaster.*^17^ Alterations in the dosage of spastin have been hypothesized to be critical for the development of the disease.^12^ A gene-dosage rescue of neurite defects has been reported in neurons differentiated from induced pluripotent stem cells derived from patients carrying nonsense mutations.^18^ In these neurons, lentiviral expression of the M1 or M87 isoforms restores para-physiological spastin levels, reducing pathological defects.^18^ Therefore, spastin-elevating strategies are emerging as promising therapeutic approaches. It has been demonstrated that spastin stability is regulated by the ubiquitin-proteasome system in a neddylation-dependent manner.^19,20^ Neddylation is a post-translational modification involving the covalent conjugation of a ubiquitin-like peptide, NEDD8, to target proteins. This modification regulates several cellular processes, including the ubiquitin-proteasome system, by affecting the stability, conformation, activity, and binding affinity of its targets.^21-24^ Inhibition of neddylation induces spastin recovery in spastin-deficient cells, in primary neurons from mice carrying heterozygous SPG4-HSP mutation, and in lymphoblastoid cell lines (LCLs) from patients carrying different haploinsufficient SPG4 mutations.^19,25^ Functionally, spastin recovery induced by inhibition of neddylation leads to the rescue of neurite defects in spastin-RNA interference (RNAi) neuronal cell models^19^ and MT cytoskeleton defects in LCLs derived from SPG4-HSP patients,^25^ showing evidence that blocking spastin poly-ubiquitination/degradation is a strategy to develop spastin-elevating therapeutic approaches. However, neddylation inhibition blocks the degradation of a large number of proteins besides spastin. In particular, broad inhibition of neddylation prevents the assembly and the activity of the large class of multi-subunit RING E3 ubiquitin ligases, known as Cullin-RING E3 ubiquitin ligases (CRLs), which regulate the degradation of approximately 20% of proteasome-regulated proteins.^23,24^

To identify a more specific strategy to block spastin degradation and assessing its impact *in vivo* in appropriate animal models, we investigated the molecular players involved in the neddylation-dependent regulation of spastin and tested them in *Drosophila melanogaster*. The main substrates of neddylation are the cullin proteins, the molecular scaffolds of the CRLs. A typical CRL consists of four subunits: a cullin (a family protein of eight members, Cul-1, 2, 3, 4a, 4b, 5, 7 or 9); a Ring-Finger protein (Roc1 or Roc2) for catalytic activity; a Cul-specific protein adaptor; and one among a plethora of diverse receptors for substrate recognition. Different subclasses of CRLs (e.g. CRL1, CRL2, etc.) can be assembled, depending on the type of cullin member engaged. Each CRL employs a unique set of substrate receptors that determine the substrate specificity of a given complex. Cullin neddylation is a key post-translational modification that triggers the assembly of CRLs and activates their E3 ligase activity, promoting poly-ubiquitination of substrates.^23,24^

Here, we identified CRL4 as a critical regulator of spastin protein levels by controlling its poly-ubiquitination and consequent degradation. We showed that downregulation or chemical inhibition of CRL4 induces recovery of spastin levels and HSP-associated phenotypes in a *Drosophila* model of SPG4 haploinsufficiency and patient-derived cells.

## Materials and methods

### Human cells, culture conditions and treatments

HeLa (a gift from N. Corbi, IBPM-CNR, Italy) and HCT116 (a gift from B. Vogelstein, Johns Hopkins University School of Medicine, Baltimore, MD, USA) cells were cultured at 37°C and 5% CO_2_ in Dulbecco’s modified Eagle medium (DMEM) GlutaMAX™ supplemented with 10% heat-inactivated fetal bovine serum (FBS) (Life Technologies, Thermo Fisher Scientific). Patient-derived lymphoblastoid cells carrying the pathogenetic haploinsufficient heterozygous c.864_865dupTC SPG4 mutation obtained as in Sardina *et al*.^21^ were cultured at 37°C and 5% CO_2_ in RPMI supplemented with 10% FBS. Cells were routinely tested for mycoplasma contamination. MG132, cycloeximide (CHX) and NSC1892/urazole were from Sigma-Aldrich (Merck). The ethics committee of ASL Roma 2, Rome, Italy, approved this study on patient-derived cells (0074975/2020). Written informed consent was obtained and data analysis was performed anonymously.

### Human expression vectors

The following plasmids were used: myc-tagged DN-Cul1; myc-tagged DN-Cul2; myc-tagged DN-Cul3; myc-tagged DN-Cul4a; myc-tagged DN-Cul5; myc-tagged wild-type Cul4a (gifts from A. Peschiaroli, IFT-CNR, Rome, Italy); and pEF-HA-Ub (gift from A. Pollice, Federico II University, Naples, Italy). Vectors were transfected by using Lipofectamine LTX and Plus reagent (Life Technologies).

### RNA interference in human cell cultures

Roc1 RNAi was carried out as described by Migita *et al*.^26^ Cul4a RNAi and DDB1 RNAi were carried out using a mix of specific validated stealth siRNAs from Life Technologies; spastin RNAi was carried out using specific stealth siRNAs selected from targeting sequences common to all spastin isoforms, as in Pisciottani *et al*.^27^ SiRNAs were transfected using Lipofectamine RNAi MAX (Life Technologies). The sequences of the siRNAs employed were as follows: (i) siCul4a #1: GAGCGGACGUUGGACAAGAUCAUGA, #2: GAGAGUGUUUCAGGAUAGACAAUAU; (ii) siDDB1 #1: UGGAGGAGCUAACUCGGAUCCAUUA, #2: AUGCAGAAUCGACUCAAUAUU; and (iii) siSpastin #1: CCAGUGAGAUGAGAAAUAUUCGAUU, #2: CGGACGUCUAUAACGAGAGUACUAA.

### Fly strains and treatments

*Drosophila* stocks and crosses were maintained on *Drosophila* standard medium (Nutri-fly, Genesee Scientific) at 25°C, unless otherwise indicated. RNAi fly lines targeting spastin expression [v108739 (P{KK107786}VIE-260B) and v33110 (w^1118^; P{GD563}v33110)] were obtained from the Vienna Drosophila Research Center (VDRC, Vienna, Austria). Bloomington Drosophila Stock Center (Indiana University, Bloomington, IN, USA) provided all the GAL4 drivers and other strains used: RNAi fly lines targeting spastin expression [27570 (y1 v1; P{TRiP.JF02724}attP2)] and [53331 (y1 sc* v1 sev21; P{TRiP.HMC03560}attP40)]; RNAi line targeting Cul4 gene [50614 ([1] v[1]; P{y[+t7.7] v[+t1.8]=TRiP.HMC02981}attP2)]; *elav*-GAL4 inserted on the X chromosome [458 (P{w[+mW.hs]=GawB}*elav*[C155]]; *eyeless-*GAL4 inserted on the second chromosome [5534 (w[*]; P{w[+m*]=GAL4-ey.H}3-8]; and GMR-GAL4 [9146 w[1118]; P{GMR-GAL4.w[-]}2/CyO]. The efficiency of each RNAi line to downregulate the expression of the corresponding target was tested by real-time reverse transcriptase (RT)-PCR and/or western blot (WB) analysis on RNAs or proteins, respectively, and extracted from different fly tissues.

For NSC1892/urazole treatment, flies were grown in water soluble Formula 4-24^®^ Instant Drosophila Medium (Carolina Biological Supply), freshly prepared at room temperature with the indicated concentrations of drug.

### Real-time RT-PCR

Total RNA was extracted using TRIzol (Life Technologies) and reverse transcribed using an iScriptTM cDNA Synthesis Kit (Bio-Rad). Real-time RT-PCR was performed using Luna^®^ Universal qPCR Master Mix (New England Biolabs) and the applied biosystem Quantstudio3 (Thermo Fisher Scientific). Relative fold-changes were determined by the 2-^ΔΔCt^ method using *giotto* or GAPDH mRNA as normalizer in flies and human cells, respectively. All reactions were performed in technical triplicate. RT-PCR was performed using dream taq polymerase (Life Technologies), and the PCR products were run on a 3% agarose gel. The spastin or cul4 amplification bands were normalized using *giotto* as the reference gene. The following primers were used: DmSpastin Forward: 5′-GAGAACGAAGGTCACAAAGAGC-3′; DmSpastin Reverse: 5′-GCTCACGCAGAGCTAGAAAATGGAG-3′; DmSpastin3 Forward: 5′-CGCTACCTATACGGTGCGAGC-3′; DmSpastin3 Reverse: 5′-AGCTCCGAAGGATGGTTCAAC-3′; DmCul4 Forward: 5′-AAGGATAAGCCTACACTGCCC-3′; DmCul4 Reverse: 5′- GTGCATCCATCTTGTGGCTAC-3′; giotto Forward: 5′-GTGCACATCGACATTGCCAACGAT-3′; giotto Reverse: 5′-TTGCAGGCCGAACCATTTGAACTC-3′; hRoc1-Forward: 5′-TTGTGGTTGATAACTGTGCCAT-3′; hRoc1-Reverse: 5′-GACGCCTGGTTAGCTTGACAT-3′; hDDB1-Forward: 5′-TAGAGATCTATGTGGTCAC-3′;hDDB1-Reverse: 5′-ATGCAGGCATTGTACTTCGCTG-3′; hGAPDH-Forward: 5′-TCCCTGAGCTGAACGGGAG-3′; hGAPDH-Reverse: 5′-GGAGGAGTGGGTGTCGCTGT-3′.

### Western blot and immunoprecipitation

Total cell extracts were prepared in lysis buffer (50 mM Tris-HCl, pH 8, 150 mM NaCl, 0.5% sodium deoxycholate, 0.1% SDS, 1% NP40 and 1 mM EDTA) supplemented with protease and phosphatase inhibitors (Roche). Proteins were resolved using Bolt Novex Bis-Tris Gels (4–12% gradient; Life Technologies). Immunoreactivity was determined using ECL-Prime (GE HealthCare); images acquisition and densitometric analysis was performed with Image Lab software (Bio-Rad). For ubiquitination assays, we first performed a WB analysis with anti-spastin on 1/10 of our immunoprecipitation (IP) reactions to determine the efficiency of IP in each sample. After this quantification, we loaded similar amounts of immunoprecipitated spastin and performed WB using anti-HA antibody (Ab).

The following Abs were employed: anti-GAPDH #sc-32233 (1:1000); anti-alpha-tubulin #sc-5286 (1:1000); anti-vinculin #73614 (1:1000); anti-Cul4a #sc-377188 (1:500); anti-p27 #sc-528 (1:200); anti-Myc #sc-40 (used to immunoprecipitate myc-Cul4a); anti-spastin mouse monoclonal Abs (1:100; sp3G11/1 #sc-53443 or sp6c6 #sc-81624; Santa Cruz Biotechnology); anti-spastin rabbit polyclonal Ab #PA5-53581 (1:1000, Life Technologies, anti-spastin monoclonal Abs were indifferently used because they produce comparable results, while anti-spastin polyclonal Ab was used in WB to detect immunoprecipitated spastin); anti-Myc #2276 (1:500, mouse monoclonal); anti-Cul4a #2699 (1:1000, rabbit polyclonal); anti-DDB1#5428 (1:1000, rabbit polyclonal) (Cell Signaling Technology); anti-HA #11583816001 (1:1000) mouse monoclonal Ab (Roche); anti-Cul2 #51-1800 rabbit polyclonal Ab (1:1000, Life Technologies); anti-horseradish peroxidase (HRP)-conjugated goat anti-mouse #7076; and anti-rabbit #7074 (Cell Signaling Technology). Normal mouse IgG #sc-2025 (Santa Cruz Biotechnology) were used as the isotype control immunoglobulins in the IP experiments.

For flies, total cell extracts were prepared in RIPA buffer (50 mM Tris-HCl, pH 7.5, 100 mM NaCl, 0.5% sodium deoxycholate, 0.1% SDS, 1% NP40, and 1 mM EDTA) supplemented with protease and phosphatase inhibitors. Proteins were quantified using the BCA protein assay kit (Pierce, Life Technologies) and then resolved by SDS-PAGE using 10% polyacrylamide gels (Bio-Rad). Immunoreactivity was determined using SuperSignal™ West Pico PLUS Chemiluminescent Substrate kit (Pierce) and image acquisition and densitometric analysis were performed using the Gel Doc XR+ Gel Documentation System and Image Lab software (Bio-Rad).

### Production of rabbit polyclonal antibody anti-Dm spastin

Rabbit polyclonal Ab production was performed by ThermoFisher Scientific. Rabbits were immunized with the Keyhole limpet haemocyanin-conjugated peptide CSLNSYEKWSQDYGDITI (Dm spastin 742:758). Serum collection was followed by a purification protocol with positive selection, with the peptide 742:758 as the immobilized antigen.

### Immunofluorescence

LCLs were seeded onto poly-L-lysine (Sigma-Aldrich) coated coverslips as in Sardina *et al*.,^21^ fixed in 2% formaldehyde, permeabilized in PBS-T (PBS 0.25% Triton-X 100) for 10 min at room temperature and then blocked in 5% bovine serum albumin (BSA) in PBS before the primary Ab (anti-β-tubulin-cy3 #C4585, Sigma-Aldrich) was applied. Nuclei were marked with Hoechst (Sigma-Aldrich). LCLs were examined under an inverted microscope (Eclipse Ti, Nikon) using a Clara camera (ANDOR Technology). Images of each sample were taken in parallel using identical microscope settings by Nis-Elements H.C. 5.11 and using the JOBS module for automated acquisitions. Analysis was performed using customized image analysis pipelines in CellProfiler, as in Sardina *et al*.^21^ The distance between cell and nucleus centroids was calculated as the length of the line segment joining two points (i.e. the nucleus and cell centroids, set to the Hoechst and beta-tubulin region of interests, respectively).

Muscle filets from *Drosophila* third instar larvae were dissected according to standard protocols.^28,29^ Briefly, larvae were dissected in PBS and fixed in 4% paraformaldehyde, 4% sucrose for 45 min at room temperature. After washes in PBS, filets were permeabilized in PBST (0.25% Triton-X 100) for 10 min at room temperature and then blocked for 1 h in blocking buffer (PBS, 0.1% Triton-X 100, 3% BSA) at room temperature, before to be incubated with primary Abs diluted in blocking buffer (PBS, 0.1% Triton-X 100, 3% BSA) for 1 h at room temperature in a wet chamber. Samples were washed with PBS and incubated with secondary Abs (Jackson ImmunoResearch) for 1 h at room temperature. After washes with PBS, preparations were mounted in antifade mounting medium with DAPI (Vectashield, Vector Laboratories). Confocal imaging was performed on a LSM700 confocal laser scanning microscope (Zeiss) equipped with four tunable light laser sources: 405, 488, 561 and 639 nm. Sequential confocal images were acquired using a ×63 oil-immersion objective with a 1024 × 1024 format, z-step size of 0.5–1 μm, with an electronic zoom magnification up to 2.0. Images were imported into ZEN 3.1 Blue Edition (Carl Zeiss Microscopy) software to obtain best fit maximum projections, which were analysed with NHI ImageJ FIJI freeware to measure the intensity of signal of the synaptic markers or to evaluate differences in larval neuromuscular junction (NMJ) morphology in different genetic backgrounds. The maximum projections were then assembled on Adobe Photoshop CS4. Quantitative analysis of NMJ markers was performed on images with a manual method based on the delimitation of pre- and postsynaptic margins, using NHI ImageJ FIJI freeware.

The following Abs were employed: anti-Discs large #4F3 (1:100), anti-Futsch #22C10 (1:100) and anti-Bruchpilot (BRP) #NC82 (1:100; Developmental Studies Hybridoma); anti-HRP FITC #123-545-021 (1:300) and anti-mouse TRITC #715-025-150 (1:300; Jackson ImmunoResearch).

### Climbing assay

Negative geotaxis assays were performed according to the standard protocols.^30^ Male adult flies were collected and placed in new vials for 24 h to eliminate the effect of CO_2_ anaesthesia. The flies were then transferred into empty vials for 1 h before being placed in the climbing assay holder. The climbing ability was scored by tapping flies to the bottom of the vial and scoring how many flies reached the target line (4 cm) after 5 s. Flies were tested in batches of 10–15, and three trials were performed on each analysis.

### Analysis of eye morphology

Pictures of fly eyes were taken at the higher magnification using the stereomicroscope (ZEISS Semi 508; 50×) equipped with an Axiocam camera 105 and exploiting ZEISS ZEN software (Blue edition 3.1). Eye area was then measured using NHI ImageJ FIJI freeware software.

### Statistical analysis

Data analyses were performed using the GraphPad Prism software 8.0.2 (GraphPad Software, Inc.). All experiments were repeated at least three times independently. To compare two samples, we determined the normality of the distribution using the Shapiro–Wilk test. For normal distributions, we used the unpaired *t*-test, otherwise, the Mann–Whitney test. For multiple comparisons, we used a one way ANOVA and Tukey's test. One way ANOVA with Dunnett's *post hoc* test were applied when two or more experimental groups were compared to a single control group. Each test was used to evaluate the statistical significance, which was set at *P* < 0.05.

## Results

### Spastin protein levels are regulated by CRL4a-mediated poly-ubiquitination

**Figure 1:**
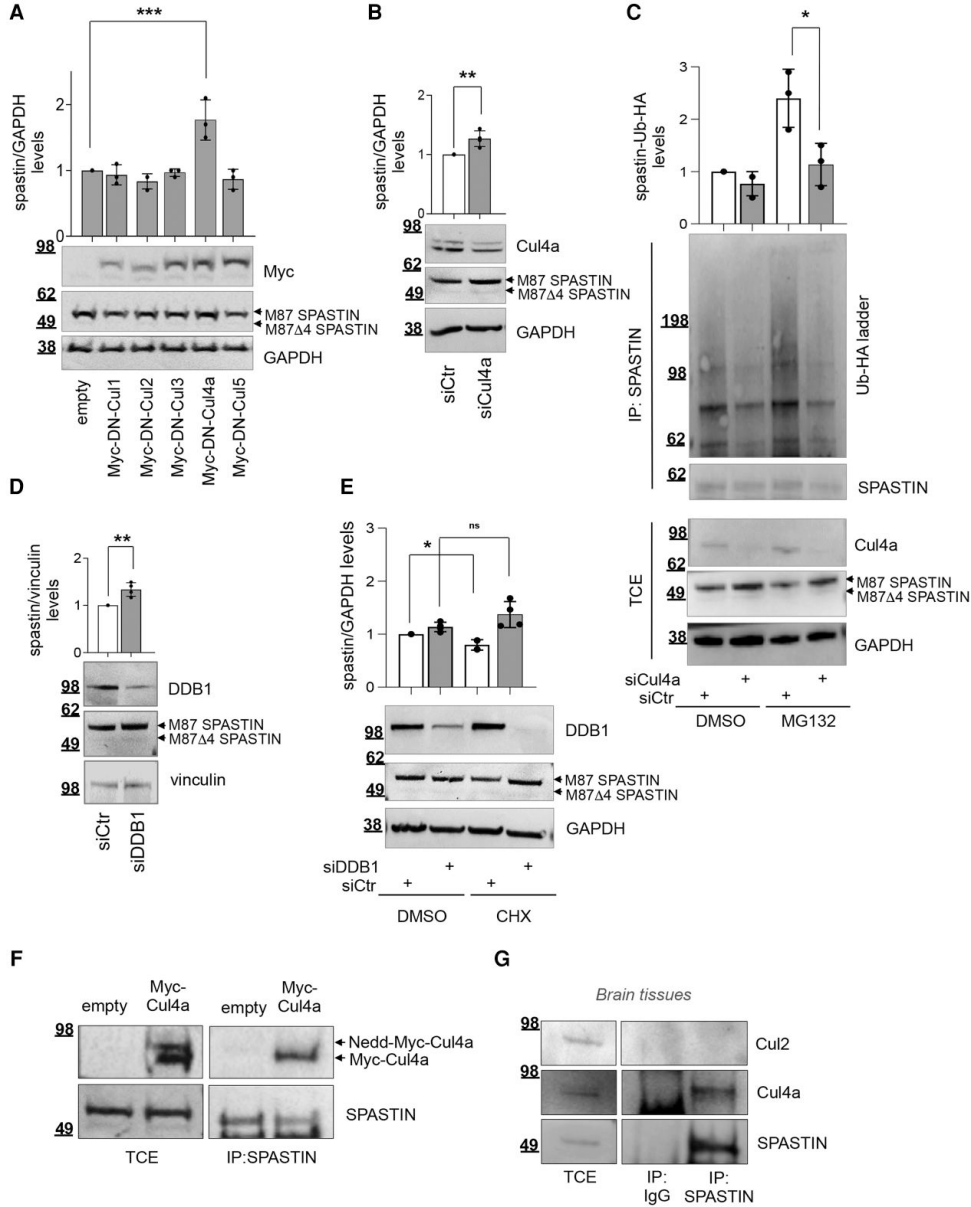
**CRLa inactivation or downregulation increases the protein levels of spastin by reducing its ubiquitination.** (**A**) Representative western blot (WB) showing increased spastin protein levels in HeLa cells 24 h after the transfection of vectors expressing the dominant-negative (DN) forms of the indicated cullins. The intensity of spastin bands was quantified and normalized by using the intensity of the GAPDH band. Quantification is reported in arbitrary units relative to control (empty vector) in the histogram as mean ± standard deviation (SD), *n* = 3. ***P < 0.001, one way ANOVA with Dunnett's *post hoc* test. (**B**) HeLa cells were transfected with a mix of Cul4a-specific (siCul4a) or negative control (SiCtr) validated stealth siRNAs and analysed by WB 48 h post transfection. A representative WB is shown. Spastin quantification is reported as mean ± SD, *n* = 4. **P < 0.01, unpaired *t*-test. (**C**) Spastin ubiquitination assays. HeLa cells were transfected with indicated siRNAs in combination with the vector expressing Ub-HA and treated with 20 μM MG132, to inhibit proteasome degradation of poly-ubiquitinated proteins, or the solvent, DMSO, 8 h before lysis. Total cell extracts (TCEs) were immunoprecipitated with anti-spastin Ab and analysed by WB 24 h post transfection. Spastin-Ub-HA ladder intensity was normalized by the intensity of spastin in the immunoprecipitation (IP) fractions and reported relative to siCtr DMSO-treated cells as mean ± SD*, n* = 3. *P < 0.05, unpaired-*t*-test. (**D**) HeLa cells were transfected with a mix of indicated validated stealth siRNA and analysed by WB 24 h post transfection. A representative WB is shown. Spastin quantification is reported as mean ± SD, *n* = 4. **P < 0.01, unpaired *t*-test. (**E**) HeLa cells were transfected as in **D** and treated with DMSO or 25 μg/ml cycloeximide (CHX) 8 h post transfection. A representative WB performed 16 h post treatment is shown. Spastin quantification is reported as mean ± SD, *n* = 4. *P < 0.05, ns = not statistically significant, unpaired *t*-test. (**F** and **G**) Representative co-IP showing Cul4a/spastin interaction *in vitro* and *in vivo*. In **F**, HeLa cells were transfected with a vector expressing Myc-tagged wild-type Cul4a or empty vector. Cells were lysed 24 h post transfection and TCEs were immunoprecipitated with anti-spastin Ab and anlysed by WB with indicated Abs. (**G**) Interaction of endogenous proteins in brain tissues. Whole brain tissues (gift from S. Soddu, IRE-IRCCS, Rome, Italy) from one adult mouse were lysed, and TCEs were immunoprecipitated with anti-spastin Ab or mouse IgG as negative isotype control and analysed by WB with indicated Abs. As a negative control, another cullin family member, such as Cul2, was included. TCE and IP samples were loaded on the same gel and processed on the same filter. Blots were vertically cropped to show appropriate expositions.

CRLs are activated by the neddylation of the cullin subunit and inhibited by the binding of Cullin-associated NEDD8-dissociated 1 (CAND1), which acts as substrate receptor exchange factor for different cullins.^24,25,31,32^ Based on our previous results showing that spastin interacts with CAND1 and is poly-ubiquitinated/degraded in a neddylation-dependent manner,^19,21^ we explored whether CRLs were involved in the regulation of spastin protein turnover. By overexpressing the dominant-negative (DN) forms of the major CAND1-controlled cullins, Cul 1–5, we found that only the expression of the DN form of Cul4a resulted in a significant increase of spastin levels in HeLa cells (Fig. 1A). Similarly, RNAi-mediated downregulation of Cul4a increases spastin levels (Fig. 1B). As reciprocal approach, we observed that the overexpression of wild-type Cul4a leads to a decrease of spastin levels (Supplementary Fig. 1A). Next, we assessed whether Cul4a regulates spastin protein levels by promoting its poly-ubiquitination by performing *in vivo* ubiquitination assays, using a vector expressing HA-tagged Ubiquitin (Ub-HA) (Fig. 1C and Supplementary Fig. 1B and C). As reported in the Fig. 1C, a significant decrease of spastin poly-ubiquitination is observed upon RNAi-mediated downregulation of Cul4a. Similar results on the poly-ubiquitination of spastin are observed by overexpressing the DN form of Cul4a (Supplementary Fig. 1B), while an increase of poly-ubiquitination is observed in cells overexpressing wild-type Cul4a (Supplementary Fig. 1C). Overall, these findings suggest that a CRL4a complex negatively regulates spastin protein levels by controlling its poly-ubiquitination and subsequent degradation.

CRL4a consists of a trimeric core (Cul4a, the unique adaptor DDB1 and the catalytic subunit Roc1), which in combination with several substrate receptors, termed DDB1-Cul4-Associated Factors (DCAFs), orchestrates poly-ubiquitination and proteasomal degradation of substrates. To corroborate the contribution of a CRL4a complex in spastin protein regulation, we tested whether RNAi-mediated downregulation of the other members of the trimeric core, i.e. DDB1 or Roc1, affects spastin levels. As reported in the Fig. 1D and Supplementary Fig. 1D, an increase of spastin levels is observed after the downregulation of DDB1 or Roc1, respectively. Accordingly, an increase in the stability of spastin is observed by treating DDB1-siRNA cells with the protein synthesis inhibitor cycloheximide (CHX) (Fig. 1E and Supplementary Fig. 1E and G). Next, we show that Cul4a interacts with spastin (Fig. 1F and G and Supplementary Fig. 1H and I). The interaction between spastin and Cul4a is shown by co-IP followed by WB analysis in cells transfected with Myc-tagged wild-type Cul4a or empty vector (Fig. 1F and Supplementary Fig. 1H and I). Furthermore, the interaction of endogenous spastin and Cul4a proteins was assessed by co-IP from murine brain lysates, showing the presence of Cul4a in spastin immunoprecipitated fraction (Fig. 1G) and supporting that spastin might form a complex *in vivo* with CRL4a in brain.

### CRL4 inhibitor NSC1892 increases spastin levels and rescues MT cytoskeleton defects in patient-derived cells

**Figure 2:**
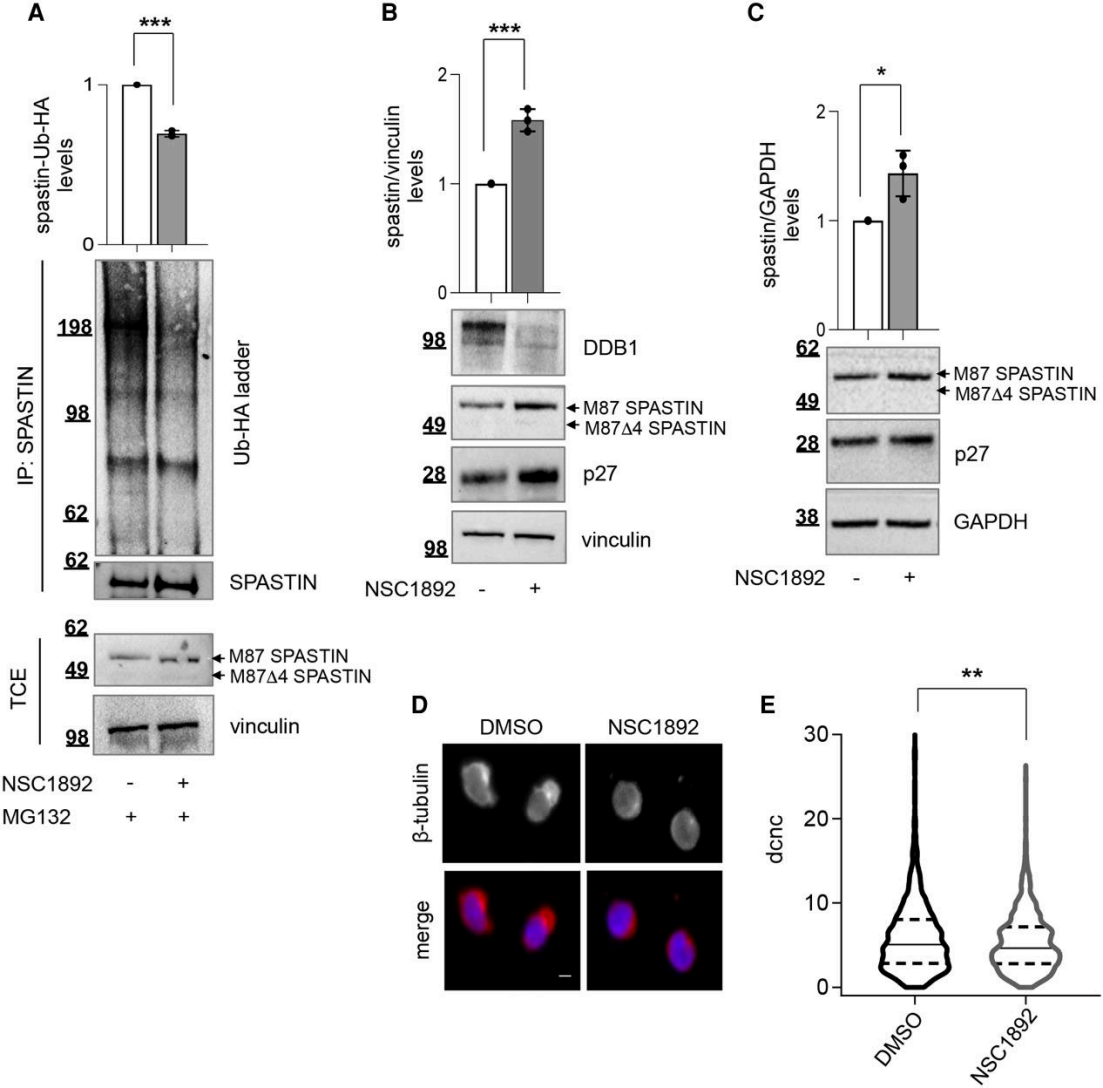
**Treatment with NSC1892 inhibits poly-ubiquitination of spastin, increases its levels and reduces defects in patient-derived cells**. (**A**) Spastin ubiquitination *in vivo* assays after NSC1892 treatment. HCT116 cells were transfected with a vector expressing Ub-HA and treated with 2 μM NSC1892 or the solvent DMSO for 21 h. MG132 was added 7 h before lysis to inhibit proteasome degradation of poly-ubiquitinated proteins. Total cell extracts (TCEs) were immunoprecipitated (IP) with anti-spastin antibody (Ab) and analysed by western blot (WB). Spastin-Ub-HA ladder intensity was normalized by the intensity of spastin in the IP fraction and reported relative to DMSO-treated cells as mean ± standard deviation (SD), *n* = 3. ***P < 0.001, unpaired *t*-test. (**B**) HCT116 cells were treated with 2 μM NSC1892 or the solvent DMSO and analysed by WB 21 h post treatment with indicated Abs. Spastin quantification is reported as mean ± SD, *n* = 3. ***P < 0.001, unpaired *t*-test. (**C**–**E**) SPG4- lymphoblastoid cell lines (LCLs) were treated with 2 μM NSC1892 or the solvent DMSO and analysed by WB or immunofluorescence (IF) 24 h post treatment. (**C**) Representative WB; p27 increase is shown as positive control for NSC1892 treatment. Data quantification relative to solvent-treatment is mean ± SD, *n* = 3. *P < 0.05, unpaired *t*-test. (**D**) Representative images of indicated cells, showing that the polarized microtubule (MT) cytoskeleton organization typical of SPG4 LCLs was rescued by NSC1892 treatment; anti-beta-tubulin Ab was used to mark the MT cytoskeleton and Hoechst staining was used for nucleus labelling. Scale bar = 10 μM. (**E**) Truncated violin plot comparing the distance between cell and nucleus centroids (dcnc) parameter. Imaging analysis was performed using a customized CellProfiler pipeline, as in Sardina *et al*.^25^ A minimum of 300 cells were analysed in three independent experiments and the grouped analysis is shown (*n* = 1380 cells treated with DMSO and *n* = 1616 cells treated with NSC1892). **P < 0.01, Mann–Whitney test. The plot reports median (dashed lines), first and third quartile (dotted lines) and density (outside lines).

To confirm the involvement of CRL4 in the regulation of spastin protein levels, we used the chemical compound NSC1892 (also known as urazole) that inhibits the interaction between DDB1 and Cul4, preventing the formation of an active CRL4 complex and causing the degradation of DDB1 and the stabilization of CRL4 targets.^33^ First, we analysed the effects of this inhibitor on spastin protein regulation. We observed a decrease of spastin poly-ubiquitination (Fig. 2A) and an increase of spastin levels (Fig. 2B) after the treatment of cells with 2 μM NSC1892. DDB1 decrease and p27 increase were shown as positive controls of the treatment (Fig. 2B). Next, we tested whether NSC1892 can induce an increase of spastin levels in a pathological SPG4 context by using patient-derived LCLs carrying SPG4-HSP heterozygous haploinsufficient truncating mutations. Differently from healthy donor LCLs, which are rounded with a central nucleus surrounded by a thin layer of MT cytoskeleton, SPG4-LCLs show a reduction of approximately 50% of spastin expression associated with a polarized organization of the MT cytoskeleton, a defect that can be rescued by increasing spastin protein levels.^25^ As shown in Fig. 2C, NSC1892-mediated inhibition of CRL4 leads to a significant recovery of spastin levels in these cells. Finally, to assess the functional effects of NSC1892-induced spastin recovery in patient cells, we analysed MT cytoskeleton organization in these cells. A cell imaging-based method that we have recently developed to quantify the polarized MT cytoskeleton of SPG4-LCLs was employed. In particular, by using this method, we have shown that SPG4-LCLs have a significant increase of the distance between cell and nucleus centroids compared to control LCLs from healthy donors.^25^ As reported in Fig. 2D and E and Supplementary Fig. 2, we observed a significant rescue of MT organization in SPG4-LCLs treated with NSC1892 compared to controls. Overall, these findings support the hypothesis that the inhibition of CRL4-mediated regulation of spastin allows its levels to be restored and reduces SPG4-HSP-associated defects in preclinical models.

### RNAi-mediated Cul4 downregulation rescues HSP-associated phenotypes in a *Drosophila* model

**Figure 3:**
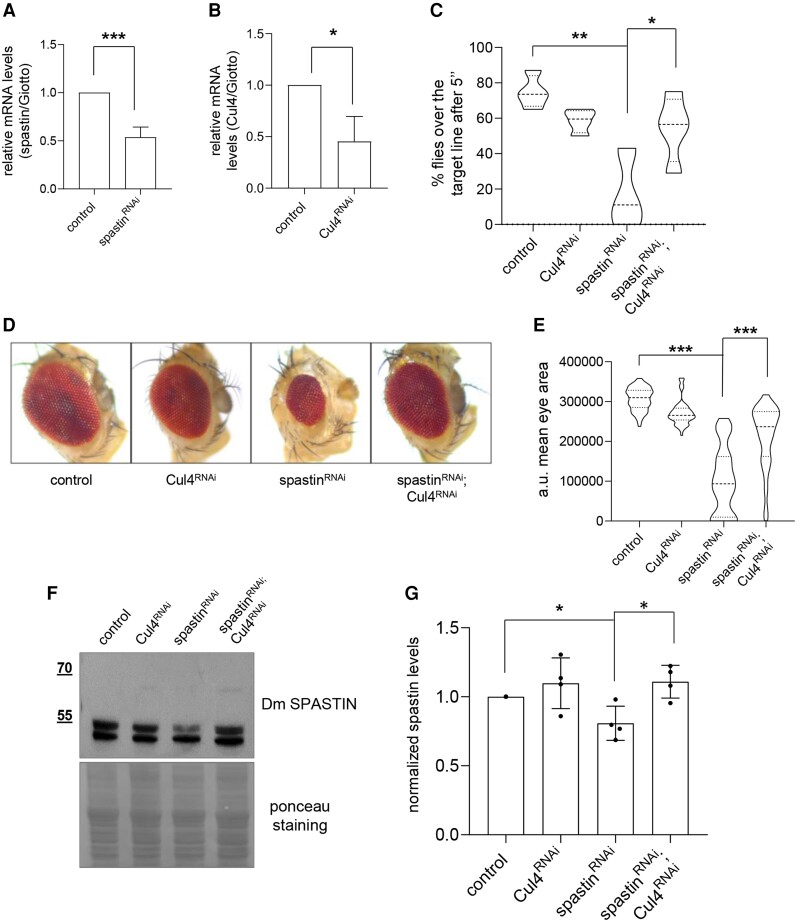
Cul4 RNAi-mediated downregulation rescues SPG4-HSP like phenotypes. (**A** and **B**) RT-PCRs on RNAs extracted from the heads of adult flies expressing the indicated RNAi constructs under control of elav-GAL4. The mRNA expression values were determined after normalization with the housekeeping gene *giotto*. ***P < 0.001 and *P < 0.05, unpaired *t*-test. (**C**) Quantification of fly locomotor activity by assessing the percentage of adult flies expressing the indicated RNAi constructs, under control of elav-GAL4, that reaches the target distance of 4 cm in 5 s (control *n* = 48 flies, Cul4-RNAi *n* = 79 flies, spastin-RNAi *n* = 37 flies, Cul4-RNAi; spastin RNAi *n* = 59 flies). The truncated violin plot reports median (dashed lines), first and third quartile (dotted lines) and density plot (outside lines). **P < 0.01 and *P < 0.05, one way ANOVA and Tukey's multiple comparison tests. (**D**) Representative images of *Drosophila* eyes from adult flies expressing the indicated constructs under control of eyeless-GAL4. Complete loss of at least one eye or a significant reduction of the eye area was observed in spastin RNAi flies. (**E**) Quantification of mean eye area for the flies with the indicated genotypes. ***P < 0.001, one way ANOVA and Tukey's multiple comparison tests. More than 50 eyes per genotype were analysed. The truncated violin plot reports all data points, median (dashed lines), first and third quartile (dotted lines) and density plot (outside lines). (**F**) Representative WB on total protein extracts obtained from fly heads expressing the indicated constructs under the control of GMR-GAL4 driver. (**G**) Spastin quantification normalized using the ponceau staining as the loading control is reported. mean ± SD *n* = 4. *P < 0.05, unpaired *t*-test. HSP = hereditary spastic paraplegia.

To functionally validate the impact of CRL4 inhibition on spastin recovery and in reducing pathological defects associated with HSP *in vivo*, we could not use mouse models, because only SPG4-homozygous knockout mice lacking spastin protein exhibit neurological disease.^34,35^ Based on the phenotypes of spastin-loss-of function *Drosophila* models, which resemble the pathological defects of HSP in human,^36-38^ we exploited a model of SPG4 haploinsufficiency in *Drosophila* by employing the GAL4-UAS system^39^ to perform RNAi-mediated downregulation of spastin in different tissues. We used a pan-neuronal driver (*elav*-GAL4) to express different UAS-spastin RNAi transgenic lines and tested their efficiency in downregulating spastin by RT-PCR. Thus, we selected the strain in which the reduction of spastin mRNA (#108739, VDRC; hereafter named UAS-spastin^RNAi^) (Fig. 3A) was not complete and compatible with a haploinsufficiency pathogenic mechanism.

Human CRL4s can contain two scaffolds, Cul4a or Cul4b, deriving from the same common ancestor and sharing many overlapping functions, while in invertebrates, such as *Drosophila*, only one highly conserved *Cul4* gene (64% identity) is present. Thus, we investigated the involvement of *Drosophila* Cul4 in spastin degradation by RNAi-mediated Cul4 downregulation. First, we confirmed the RNAi-mediated downregulation of Cul4 by RT-PCR (Fig. 3B). Flies expressing UAS-spastin^RNAi^ in all neurons showed an unexpanded wing phenotype (Fig. 3C–E). Next, we assessed the locomotor behaviour of these flies. A climb assay revealed that spastin-RNAi flies have a strong impairment of locomotor ability (Fig. 3C). Similar results were also obtained in other lines expressing different UAS-spastin RNAi (Supplementary Fig. 3A and B), according to different fly models carrying spastin mutations, and resembling human motor deficits observed in SPG4-HSP patients.^17,34-36^ Indeed, only a small percentage of spastin-RNAi flies reached the target point in the defined time. Downregulating Cul4 significantly rescued the climbing defects in these flies (Fig. 3C).

In addition, we assessed the effects of spastin downregulation on fly eye development as a general model of neurodegeneration by expressing the RNAi constructs early or late during eye development. Most of the flies expressing UAS-spastin ^RNAi^ early during eye development (*eyeless*-GAL4 driver^40^) did not develop eyes at all or showed a significant reduction of the eye area (Fig. 3D). Similar results were also obtained in other lines expressing different UAS-spastin RNAi, supporting the specificity of the eye phenotype (Supplementary Fig. 3C and D), consistent with the small eyes observed in zebrafish spastin mutants.^41^ Interestingly, the eye area was significantly rescued when both spastin and Cul4 were downregulated by RNAi (Fig. 3D and E). We expressed the RNAi constructs under the control of glass multiple reporter (GMR-GAL4 driver), which expresses late during eye development and posteriorly to the morphogenetic furrow.^42^ Thus, we confirmed that Cul4 downregulation suppresses retina degeneration due to spastin RNAi-mediated downregulation (Supplementary Fig. 4A–C). In particular, we calculated the degree of degeneration, measuring the extension of the depigmented area of each eye (Supplementary Fig. 4B).

Finally, to verify the effects of Cul4 downregulation on spastin recovery, we performed a WB analysis from fly head extracts. Since a commercial Ab recognizing *Drosophila* spastin was not available, we generated a custom polyclonal Ab against a synthetic peptide of *Drosophila* spastin. According to the results obtained *in vitro* (Fig. 1 and Supplementary Fig. 1), spastin protein levels are significantly restored by Cul4 downregulation in spastin RNAi flies (Fig. 3F and G).

### Cul4 inhibition rescues NMJ morphology, recovers spastin levels and reduces HSP defects *in vivo*

**Figure 4:**
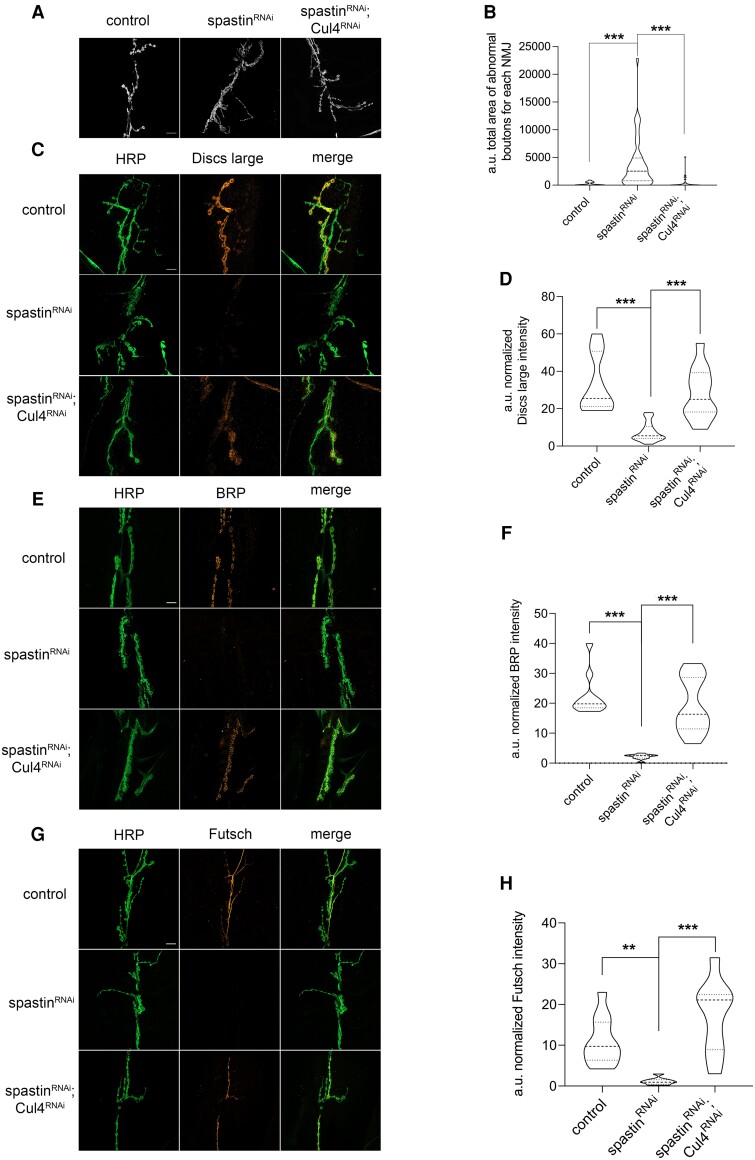
**Spastin RNAi affects neuromuscular junction morphology and Cul4 downregulation significantly rescues these alterations.** (**A**–**H**) Third instar larva neuromuscular junctions (NMJs) on muscles 6 and 7 of flies expressing the indicated constructs under control of elav-GAL4 driver were analysed by immunofluorescence (IF). (**A**) Representative NMJ images after staining with anti-horseradish peroxidase (HRP) to highlight presynaptic membranes. Bouton morphology changed when spastin was downregulated but was significantly restored by Cul4 RNAi. (**B**) The area of all boutons of each NMJ was measured. The area of each abnormal bouton that was above a fixed threshold (area >1000 a.u.) was considered and summed. The total area occupied by all abnormal boutons of each NMJ was normalized for the length of all NMJ branches. At least 18 NMJs for each genotype were analysed (control *n* = 18 NMJs, spastin-RNAi *n* = 44 NMJs and spastin-RNAi;Cul4-RNAi *n* = 34 NMJs). ***P < 0.001, one way ANOVA and Tukey's multiple comparison test. (**C**) NMJs were co-stained with anti-HRP and anti-Discs large, a marker of subsynaptic reticulum. (**D**) Discs large signal intensity, after background subtraction, was measured and divided by NMJ total area for normalization. At least 18 NMJs for each genotype were analysed (control *n* = 18 NMJs, spastin-RNAi *n* = 44 NMJs and spastin-RNAi;Cul4-RNAi *n* = 34 NMJs). ***P < 0.001, one way ANOVA and Tukey's multiple comparison test. (**E**) NMJs were co-stained with anti-HRP and anti-BRP, which labels the T-bar specialization at the active zone. (**F**) BRP signal intensity, after background subtraction, was measured and divided by NMJ total area for normalization. At least 10 NMJs per genotype were analysed (control *n* = 10 NMJs, spastin-RNAi *n* = 10 NMJs and spastin-RNAi/Cul4-RNAi *n* = 15 NMJs). ***P < 0.001, one way ANOVA and Tukey's multiple comparison test. (**G**) NMJs were co-stained with anti-HRP and anti-Futsch, which labels a presynaptic microtubule (MT)-associated marker. (**H**) Quantification of Futsch signal intensity. After background subtraction, signals were divided by NMJ total area for normalization. At least eight NMJs per genotype were analysed (control *n* = 12 NMJs, spastin-RNAi *n* = 8 NMJs, and spastin-RNAi;Cul4-RNAi *n* = 9 NMJs).**P < 0.01, ***P < 0.001, one way ANOVA and Tukey's multiple comparison test. All crosses for the NMJ analysis were held at 29°C. Scale bars = 10 μm. The truncated violin plots report median (dashed lines), first and third quartile (dotted lines) and density plot (outside lines).

Considering the beneficial impact of Cul4 downregulation on spastin levels and locomotor defects, we assessed its effect on the morphology of larval NMJs^43,44^ of muscles 6 and 7. Third instar larva NMJs from flies expressing either UAS-spastin^RNAi^, UAS-Cul4^RNAi^ or both were labelled with anti-HRP, which stains presynaptic neuronal membrane and allows synaptic bouton morphology to be studied. In flies expressing UAS-spastin^RNAi^, we observed an increase in the area of abnormal boutons, which was restored by Cul4 downregulation (Fig. 4A and B). Next, we assessed whether morphological alterations of synaptic boutons were associated with the mislocalization of synaptic markers. NMJs were co-stained with anti-HRP and anti-Discs large, which labels the postsynaptic specialization named the subsynaptic reticulum. RNAi-mediated downregulation of spastin reduced the postsynaptic level of Discs large,^45,46^ which was significantly recovered by Cul4 downregulation (Fig. 4C and D). During the embryonic development of NMJs, after axonal growth cones establish contacts with target muscles, NMJs differentiate presynaptic terminals that promote the clustering of postsynaptic adaptor proteins. Since NMJ formation relies on an intimate temporally regulated interaction between pre- and postsynaptic specializations, we further analysed NMJs by assessing whether the presynaptic side of the active zones was affected by spastin downregulation and recovered by Cul4 downregulation. We labelled NMJs with anti-BRP, which stains the presynaptic specializations named T-bars. UAS-spastin^RNAi^ flies showed a reduction in the BRP signal intensity that was significantly recovered by Cul4 downregulation (Fig. 4E and F). Finally, since neuronal MT distribution has been reported to be defective at the NMJs of other SPG4-HSP fly models,^36-38,47^ we assessed the MT distribution in our UAS-spastin^RNAi^ flies by labelling NMJs with an Ab recognizing Futsch, the fly orthologue of mammalian MT-associated protein 1 (MAP1). We observe a decrease in Futsch levels in spastin RNAi flies compared to the control, which was significantly restored by Cul4 downregulation (Fig. 4G and H).

**Figure 5:**
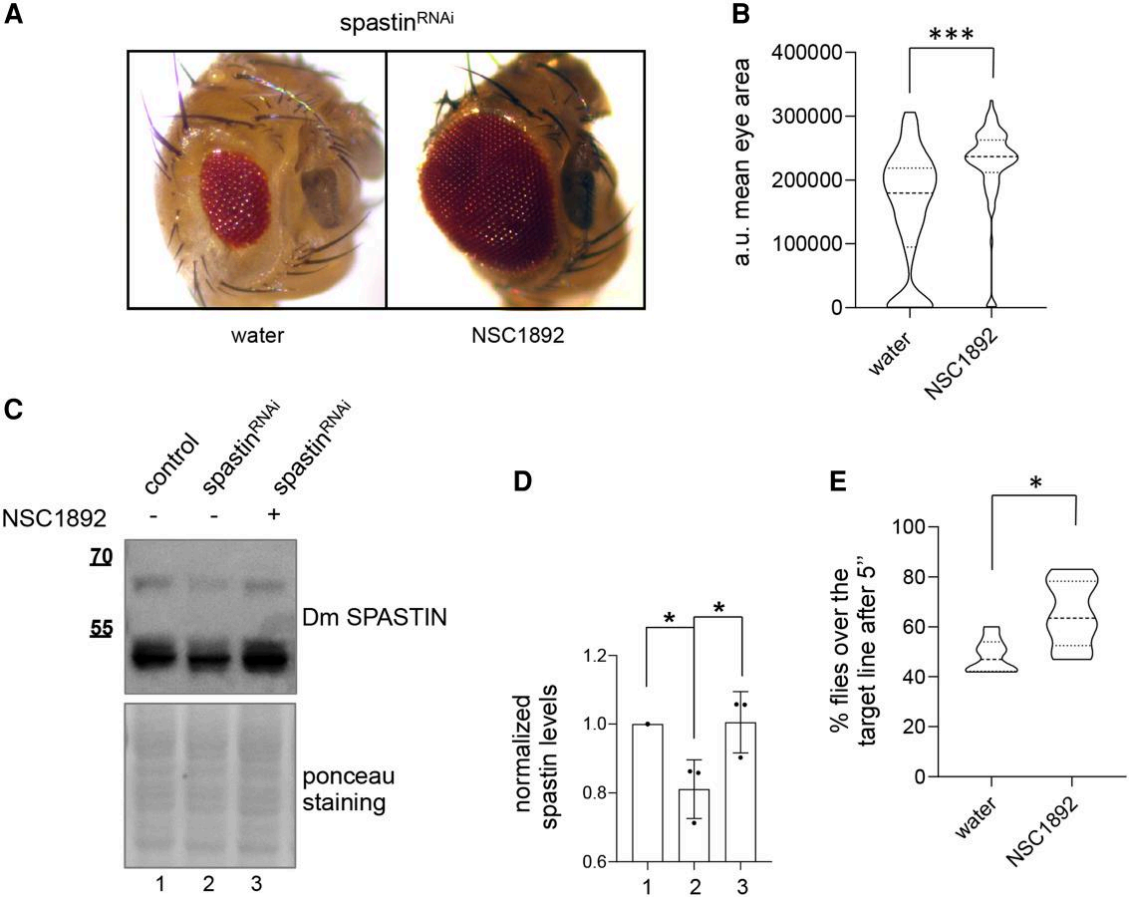
**Growing flies in food supplemented with NSC1892 increases spastin levels and rescues defects in fly model of SPG4-HSP**. (**A**) Representative images of flies expressing UAS-spastin^RNAi^ early during eye development (eyeless-GAL4) grown with food containing 3 μM NSC1892 or water. Note that differently from *in vitro* experiments, NSC1892 powder was freshly prepared in water for all *in vivo* assays. (**B**) Quantification of the eye area of flies corresponding to each treatment in three independent experiments. Grouped analysis of eye area mean was shown for *n* = 174 flies grown in water supplemented food and *n* = 102 flies grown in NSC1892-supplemented food. ***P < 0.001 Mann–Whitney test. (**C**) Representative western blot on total protein extracts obtained from indicated flies grown with food containing 3 μM NSC1892 or water. (**D**) Spastin quantification performed and reported as in Fig. 3I; mean ± standard deviation, *n* = 3. *P < 0.05 one way ANOVA and Tukey's multiple comparison test. (**E**) Locomotor activity of flies expressing UAS-spastin^RNAi^ under control of elav-GAL4 driver, grown with food containing 3 μM NSC1892 or water is quantified and reported as in Fig. 3F, in three independent experiments. Grouped analyses of more than 100 flies per condition are shown. *P < 0.05, unpaired *t*-test. The truncated violin plots report median (dashed lines), first and third quartile (dotted lines) and density plot (outside lines). HSP = hereditary spastic paraplegia.

Finally, as a proof of concept of a pharmacological treatment inhibiting CRL4, we tested the effects of NSC1892 *in vivo* in UAS-spastin^RNAi^ flies. We grew flies expressing UAS-spastin^RNAi^ early during eye development (*eyeless*-GAL4) in food containing increasing doses of NSC1892. At the concentration of 3 μM NSC1892 we observe a significant recovery of the eye area (Fig. 5A and B and Supplementary Fig. 5A) and a significant increase in spastin protein levels (Fig. 5C and D). Similarly, flies expressing UAS-spastin^RNAi^ under the control of the pan-neuronal driver (*elav*-GAL4) and grown with food containing NSC1892 significantly improved their locomotion activity (Fig. 5E and Supplementary Fig. 5B), confirming that inhibition of the CRL4 core complex restores spastin levels and reduces SPG4-HSP defects *in vivo*.

## Discussion

HSP is a motor neuron neurodegenerative disorder commonly associated with SPG4 gene haploinsufficiency. Strategies to induce a recovery of spastin protein levels are considered novel promising therapeutic approaches. In the past, we have shown that spastin levels are regulated by neddylation-dependent poly-ubiquitination and proteasomal-mediated degradation. Here, we demonstrated that the CRL4 complex regulates spastin stability. In particular, we show that spastin levels are regulated by CRL4a-mediated poly-ubiquitination and that spastin interacts with the CRL4 complex (Fig. 1). To confirm these findings, we showed similar results in spastin-proficient cells and in the SPG4 pathological context by chemical inactivation of the CRL4 core complex using NSC1892. We demonstrated that NSC1892 induces a spastin recovery and rescues defects of MT organization in patient-derived cells (Fig. 2). To functionally validate the effects of CRL4 inhibition *in vivo*, we exploited a *Drosophila* model of SPG4 haploinsufficiency, through RNAi-mediated downregulation of spastin in various fly tissues. We observed that Cul4 RNAi-mediated downregulation significantly rescues HSP-like phenotypes due to spastin RNAi (Fig. 3). Furthermore, we demonstrated that Cul4 downregulation rescues alterations of NMJ morphology due to spastin RNAi (Fig. 4). Finally, we showed the increase of spastin levels and the reduction of HSP-like defects in flies supplemented with NSC1892. This provides the proof of concept that a pharmacological treatment inhibiting CRL4 might represent a new spastin recovery therapeutic approach (Fig. 5).

In cells from patients, we measured the functional effects of CRL4 inhibition by using a fast automated non-invasive cell imaging method, opening the possibility to generate outcome measures useful for future clinical trials based on spastin recovery approaches. In addition, we observed that any treatment employed to silence or inhibit CRL4 in cell cultures or flies did not induce any major accumulation of spastin, despite prolonged expression of RNAi in flies, exposure of cells to high levels of siRNAs or high doses of NSC1892 inhibitor. This supports the feasibility of spastin recovery therapeutic approaches targeting the CRL4 complex, which does not produce any toxic effects associated with spastin overdosage. This also suggests that the regulation of spastin protein stability does not fully rely on CRL4 and supports the hypothesis that spastin levels are very finely tuned to accurately avoid its overdosage. It has been demonstrated that small differences in the expression level of wild-type spastin generate significant functional consequences and that the upregulation of spastin activity in neurons produces deleterious effects on neurite morphology and dynamics.^9,12^ This supports the idea that different pathways may cooperate to reduce the stability of spastin, preventing its overdosage, even in the context of SPG4 haploinsufficiency.

An important criticism of the idea to develop novel spastin-elevating approaches to treat SPG4-HSP is the limitation of using these treatments only for patients carrying SPG4 haploinsufficient mutations. It seems that it is not useful to design spastin-elevating approaches for patients carrying missense mutations. However, with the increasing knowledge of antisense oligonucleotide (ASO)-mediated therapies for the treatment of rare neurological disorders, it is possible to envisage the combination of specific ASOs, which can reduce the sole expression of the mRNAs carrying spastin missense mutations, with spastin-elevating approaches to increase the post-translational stability of the wild-type variant.

*Drosophila* has been used successfully as an *in vivo* model system to dissect the functional role of spastin in the regulation of NMJ development and activity.^36-38,47^ Different alleles and genetic tools have been exploited including classical null alleles, single amino acid substitutions and transgenic lines expressing RNAi constructs to target spastin mRNA. These tools have enabled researchers to demonstrate that spastin loss in *Drosophila* causes alterations in the synaptic development of NMJs and defects in neurotransmission. In particular, it has been observed that the morphological and functional defects are associated with an aberrant stabilization of the MT cytoskeleton at the pre-synapse. Although there is total agreement in the literature in assigning spastin a central role in the regulation of MT dynamics in the axons and at the synapse, the phenotypes observed in each case underlined slight differences. It has been observed that RNAi-mediated downregulation of spastin, as well as the expression of spastin missense mutations, causes the undergrowth of NMJ and a specific reduction in the synaptic area.^37,38^ Conversely, a prominent increase in the number of boutons associated with alterations in the synaptic structure and formation of abnormal grape-like clusters has been observed in different spastin classical null alleles.^36,47^ The appearance of bouton clusters was also linked to the lack of distal MTs. In agreement with these observations, we found that flies expressing a specific RNAi construct, which reduces spastin mRNA levels by 50%, under control of the pan-neuronal driver *elav*-Gal4, show the formation of abnormal boutons resembling those characterized by a grape-like structure (Fig. 4). Furthermore, coherently with alterations in neurotransmission, we observed that adult flies expressing spastin RNAi under *elav*-Gal4 control show a significant reduction in locomotion activity. Defects in neurotransmission and locomotion activity are consistent with reduced levels of pre- and postsynaptic markers, such as BRP and Discs large, and the MT-associated protein Futsch (Fig. 4). Beyond NMJ defects, we observed typical locomotion and eye phenotypes in four different UAS-spastin RNAi lines, according to literature.^36-38,41^ Additionally, we observed that flies expressing one spastin RNAi under *elav*-Gal4 control show an unexpanded wing phenotype, which significantly ameliorates upon Cul4 downregulation (Supplementary Fig. 6). However, as this unexpanded wing phenotype was observed only in one of the four different UAS-spastin RNAi analysed lines, it cannot be assumed to be a specific effect due to spastin RNAi without further exploration. Nevertheless, it remains intriguing, since similar alterations of wing development has been also reported in diverse fly models of motor neuron degeneration.^48,49^

In recent years, there has been an emerging interest in the identification of inhibitors of the trimeric CRL4 core complex (e.g. NSC1892,^33^ TSC1682,^50^ 33-11 and KH-4-43^51^). The development of these inhibitors, as well as neddylation inhibitors, stems from the observation that overexpression of cullins has been reported in several types of cancer.^52^ We have previously observed that the inhibition of neddylation promoted spastin stability.^19^ As positive regulation by neddylation is a general pathway shared by several cullins, we searched for a more specific cullin-mediated pathway that controls spastin stability and demonstrated an important role played by CRL4. Although the data presented here underline a specific role of CRL4 in spastin stability, we cannot exclude that other CRLs or other degradative processes may contribute to the control of spastin homeostasis.

To achieve a very specific recovery of spastin protein levels, it will be important to identify the DCAFs of the CRL4/spastin complex. For example, thalidomide and its derivatives have been developed to target cereblon, a DCAF of CRL4, for the treatment of multiple myeloma and other malignancies.^53,54^ Although further experiments are necessary to identify which DCAFs specifically recruit spastin for poly-ubiquitination, we can now capitalize on the evidence provided on the role of CRL4 to identify those substrate receptors that work together with CRL4 in the poly-ubiquitination of spastin. Thus, by deciphering the complex regulatory mechanisms of spastin and exploiting the druggability of CRL4, we can pave the way for novel therapeutic interventions, offering new hope for improved clinical outcomes in SPG4-HSP patients.

## Supplementary Material

awae095_Supplementary_Data

## Data Availability

Additional data, material, and protocols are provided upon reasonable request to the corresponding author.
