# Supplementary material for: Cul-4 inhibition rescues spastin levels and reduces defects in hereditary spastic paraplegia models
Source: Brain. 2024 Mar 29;147(10):3534–46. doi: 10.1093/brain/awae095 (PMC11449140; doi:10.1093/brain/awae095)
Supplement: awae095_Supplementary_Data [file awae095_supplementary_data.zip › brain-2023-01619-File007.pdf]

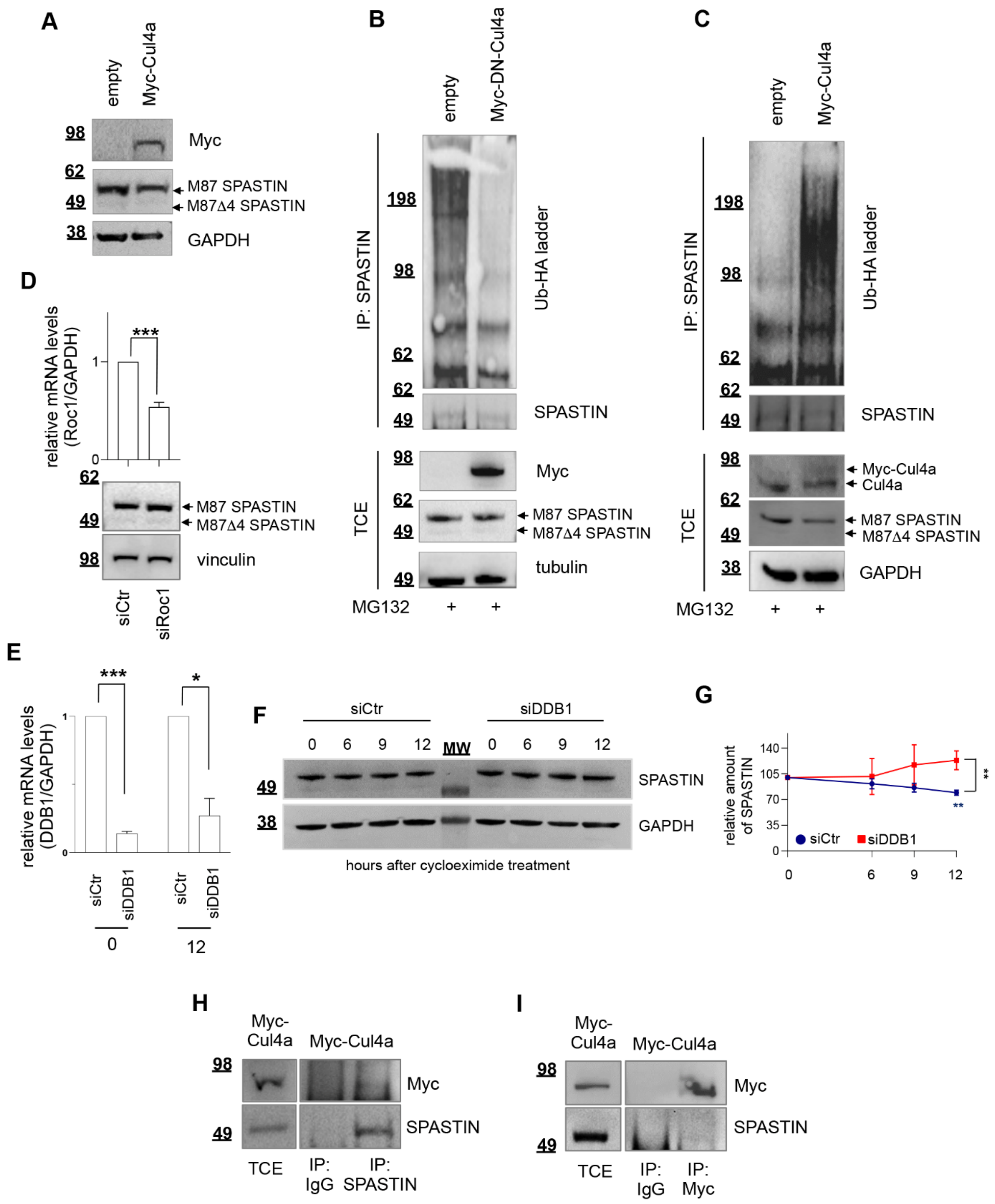

Supplementary Figure 1. CRL4a regulates spastin protein levels by poly-ubiquitination mediated degradation. (**A**) WB showing decreased spastin protein levels 24h after Myc-tagged Cul4a expression in HeLa cells. (**B-C**) Spastin ubiquitination assays. HeLa cells were transfected with indicated expressing vectors in combination with a vector expressing Ub-HA, and treated with 20µM MG132, to inhibit proteasome degradation of poly-ubiquitinated proteins, 8h before the lysis. TCE were immunoprecipitated with anti-spastin Ab and analysed 24h post transfection by WB with indicated Abs. (**D**) HeLa cells were transfected with indicated Roc1 siRNAs and analysed 48h post transfection by WB to analyze spastin levels (low panels) and real time RT-PCR to verify Roc1

downregulation (top panel). The mRNA expression values were determined after normalization with the housekeeping gene GAPDH. *n=3* technical replicates. ****P<0.001,* unpaired t-test. (**E-G**) HeLa cells were transfected and treated as in 1E, and analysed by real time RT-PCR to verify DDB1 downregulation (**E**) and by WB (**F**) at the indicated hours post treatment. In **G**, WB quantification; the levels of spastin relative to those of loading control were measured at each time point and reported as mean ± SD, *n=3* independent experiments. Differences among spastin protein levels in siCtr cells were calculated and a significant decrease of spastin levels at 12h post treatment (12) was observed compared to untreated cells (0), ***P<0.01* (represented in blue), unpaired t-test. Differences between siCtr and siDDB1 cells were calculated for each time point, showing a slower clearance of spastin levels in siDDB1i compared to siCtr cells, ***P<0.01*, unpaired t-test. (**H-I**) HeLa cells were transfected with a vector expressing Myc-tagged wild-type Cul4a. In **H**, cells were lysed 24h post transfection and TCE were immunoprecipitated with anti-spastin Ab or IgGs as negative control and anlysed by WB with indicated Abs. In **I**, cells were lysed 24h post transfection and TCE were immunoprecipitated with anti-Myc Ab or IgGs as negative control and anlysed by WB with indicated Abs. TCE and IP samples were loaded on the same gel and processed on the same filter. Blots were vertically cropped to show appropriate expositions and exclude unrelated lanes.

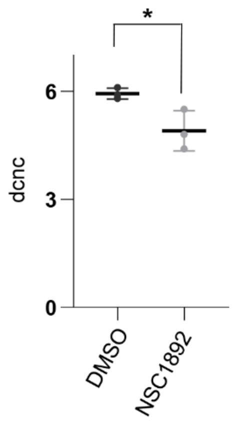

Supplementary Figure 2. Inter-experimental variability of the dcnc parameter in response to NSC1892 treatment in SPG4-HSP patient-derived cells. The dcnc measurements of the experiments shown in the Figure 2E are reported as mean ± SD for *n=3* independent experiments. **P<0.05*, unpaired t-test.

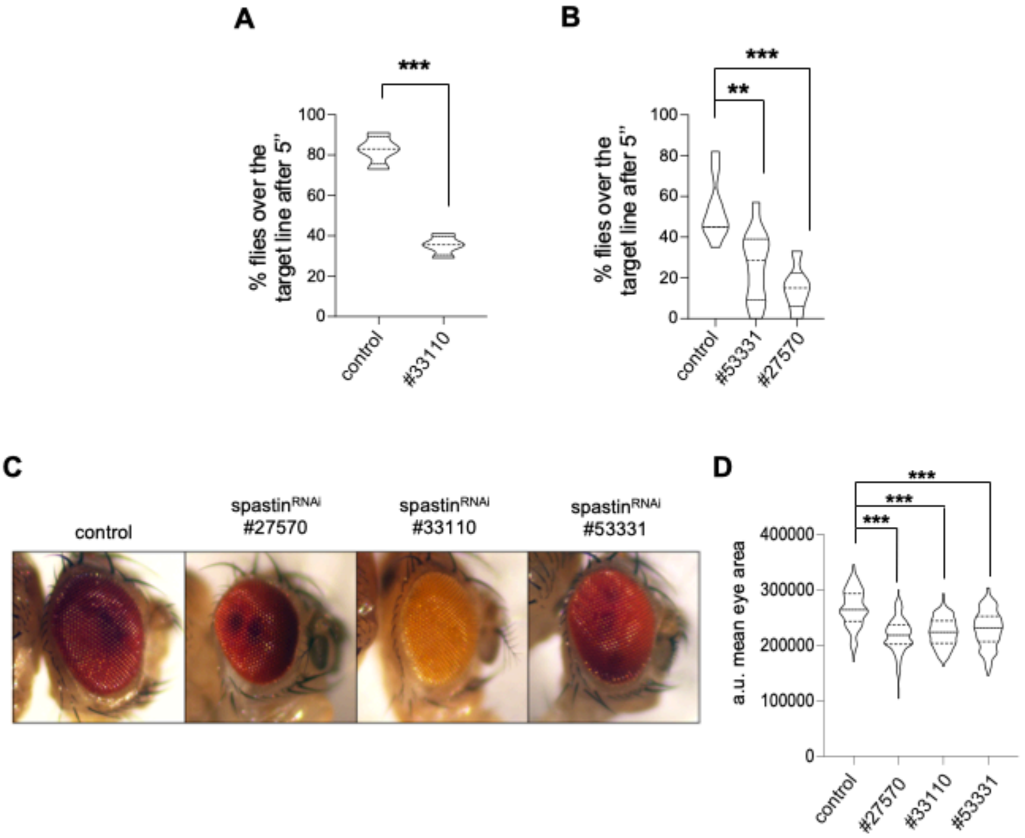

Supplementary Figure 3. Locomotor and eye phenotypes in flies expressing different spastin-RNAi constructs. (**A-B**) Percentage of adult flies, expressing the indicated spastin-RNAi construct under control of *elav*-GAL4, which reaches the target distance of 4 cm in 5 seconds. In **A**, control *n=96* flies; #33110, *n=110* flies; in **B**, control *n=42* flies; #53331 *n=62* flies; and #27570 *n=29* flies. Normal distribution was confirmed by Shapiro-Wilk test; the unpaired t test was performed in **A**, and one way ANOVA with Dunnett's post hoc test in **B**. ***P<0.01, ***P <0.001*. Truncated violin plots report median (dashed lines), first and third quartile (dotted lines) and density plot (outside lines). (**C**) Representative images of Drosophila eyes from adult flies expressing the indicated spastin-RNAi constructs, under the control of *eyeless*-GAL4. (***D***) Quantification of the mean eye area of the indicated flies. More than 130 eyes per genotype were measured (control *n=154* eyes; #27570 *n=159* eyes; #33110 *n=130* eyes, and #53331 *n=137* eyes). One way ANOVA with Dunnett's post hoc test ****P <0.001*. Truncated violin plot reports median (dashed lines), first and third quartile (dotted lines) and density plot (outside lines).

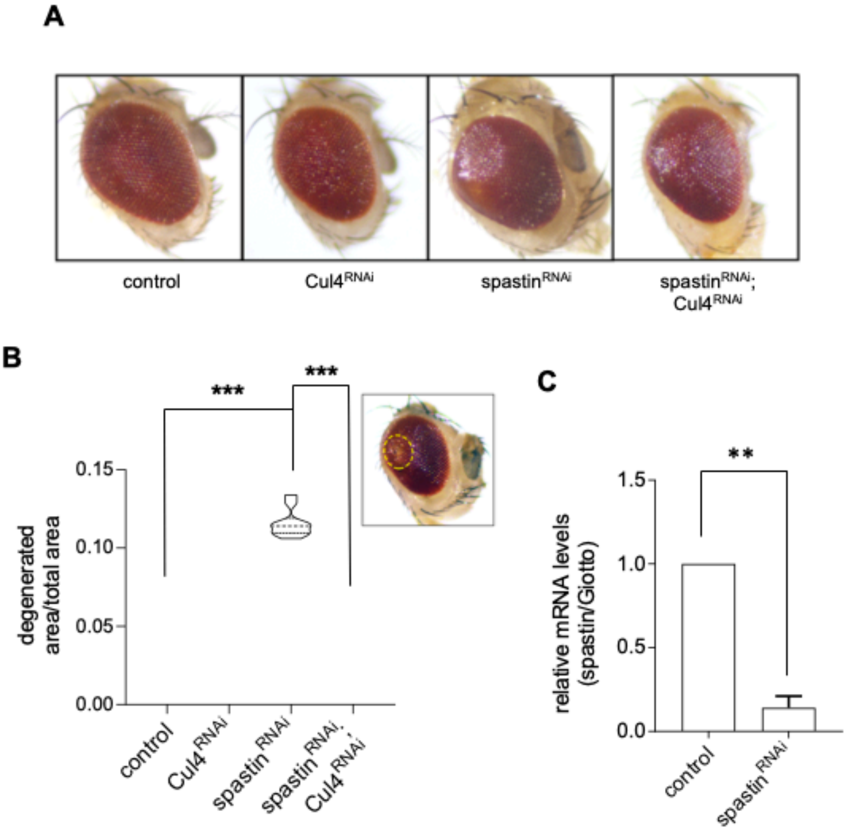

Supplementary Figure 4. Cul4 RNAi-mediated suppression of retina degeneration. (**A-B**) In **A**, representative images of Drosophila adult eyes expressing the indicated RNAi constructs under control of GMR-GAL4 driver. In B, quantification of the depigmented eye area as index of degeneration. The inset panel shows a representative image of a spastin RNAi eye, the area in the circle outlined by the dotted yellow line is the depigmented region under consideration. Control *n=23* eyes, Cul4-RNAi *n=35* eyes, spastin-RNAi #108739 *n=10* eyes, Cul4-RNAi;spastin-RNAi #108739 *n=23* eyes. ****P<0.00*1, one way ANOVA and Tukey's multiple comparison test. Truncated violin plot reports median (dashed lines), first and third quartile (dotted lines) and density plot (outside lines). (C) Real-time RT-PCR on RNAs extracted from the heads of adult control flies and flies expressing the spastin-RNAi #108739 construct under control of GMR-GAL4, mean ± SD, n=3 technical replicates, ***P<0.01*, unpaired t-test.

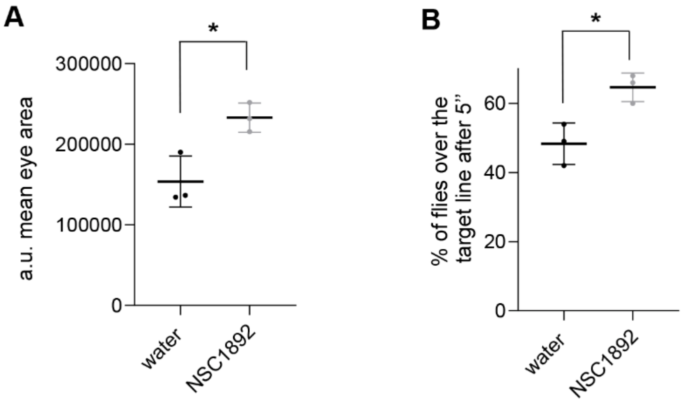

Supplementary Figure 5. **Inter-experimental variability of the effects of NSC1892 treatment on fly model.** (**A**) The eye area of the experiments shown in the Figure 5B are reported as mean ± SD for *n=3* independent experiments. **P<0.05*, unpaired t-test. (**B**) The percentage of locomotor activity of the experiments shown in the figure 5E are reported as mean ± SD for *n=3* independent experiments. **P<0.05*, unpaired t-test.

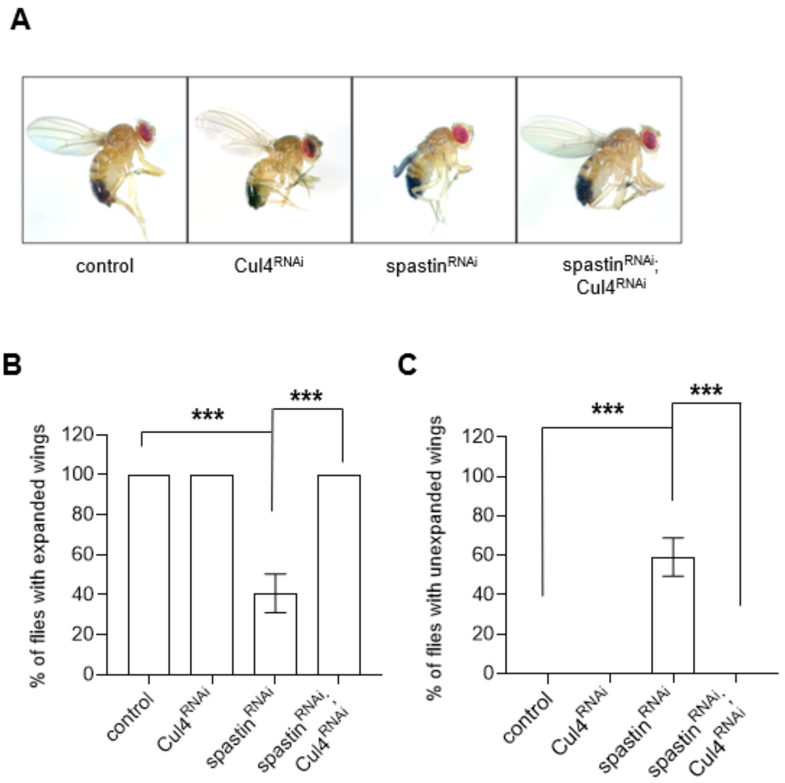

Supplementary Figure 6. **Defects in wing expansion in flies expressing the spastin-RNAi #*108739* construct under control of *elav*-GAL4.** (**A**) Representative images of Drosophila adult flies expressing the indicated RNAi constructs under control of *elav*-GAL4. (**B-C**) The percentage of flies with expanded (**B**) and unexpanded (**C**) wings for each genotype. More than 120 flies per genotype were analysed and reported as mean ± SD ****P<0.001*, one way ANOVA and Tukey's multiple comparison test.
