# Supplementary material for: Cul-4 inhibition rescues spastin levels and reduces defects in hereditary spastic paraplegia models
Source: Brain. 2024 Mar 29;147(10):3534–46. doi: 10.1093/brain/awae095 (PMC11449140; doi:10.1093/brain/awae095)

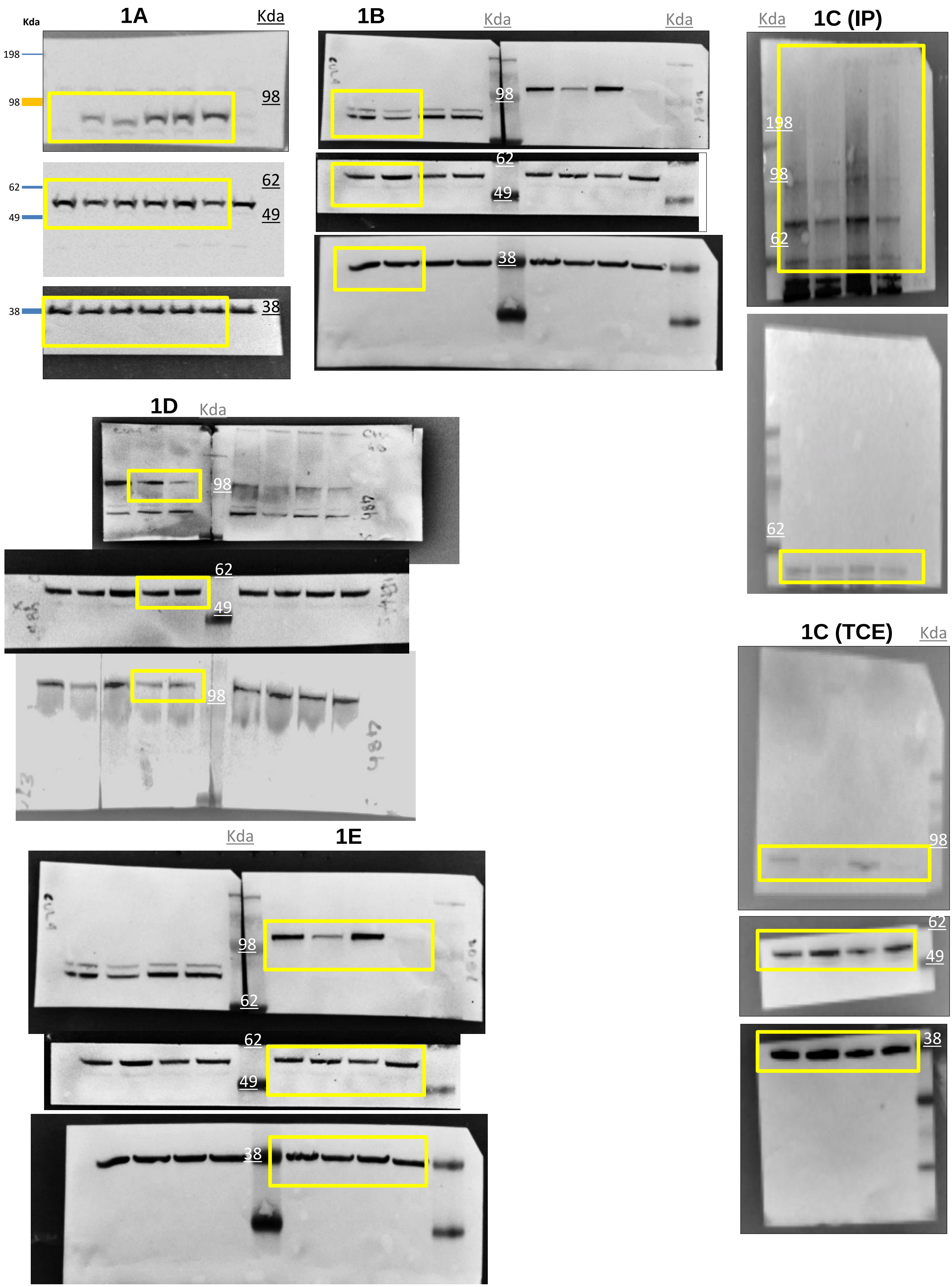
1A
Kda
198
98
62
49
38
1B
1C (IP)
1C (TCE)
1D
1E

1F (TCE)

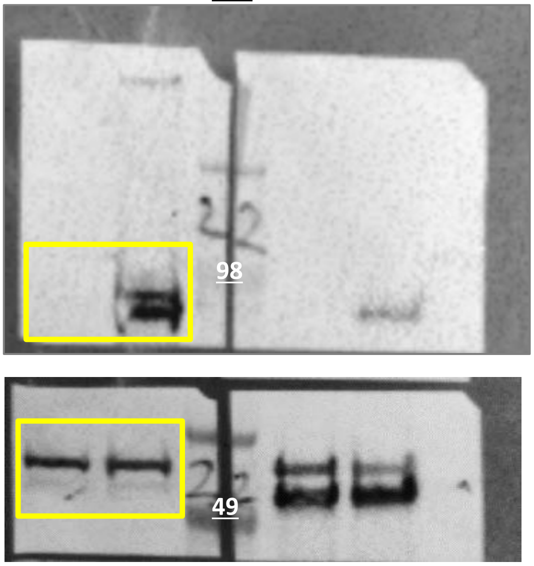

1F (IP)

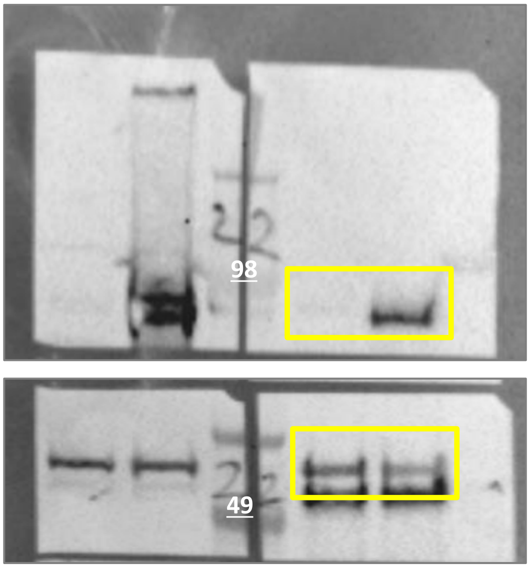

1G (TCE)

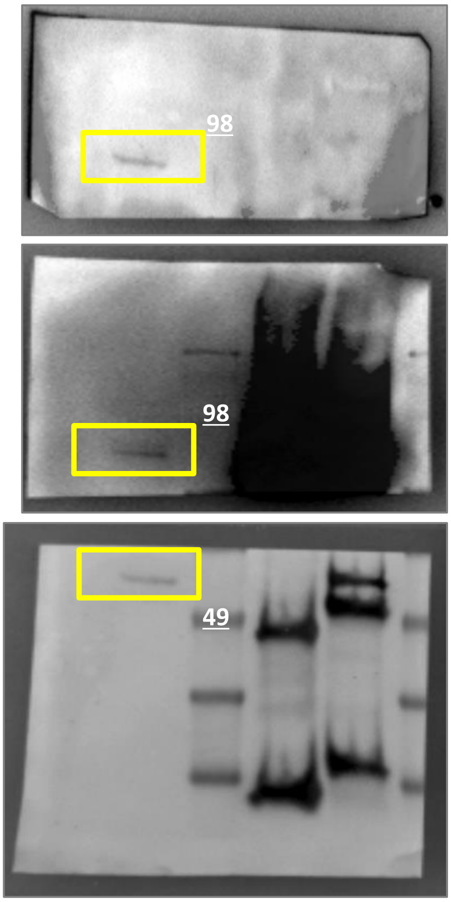

1G (IP)

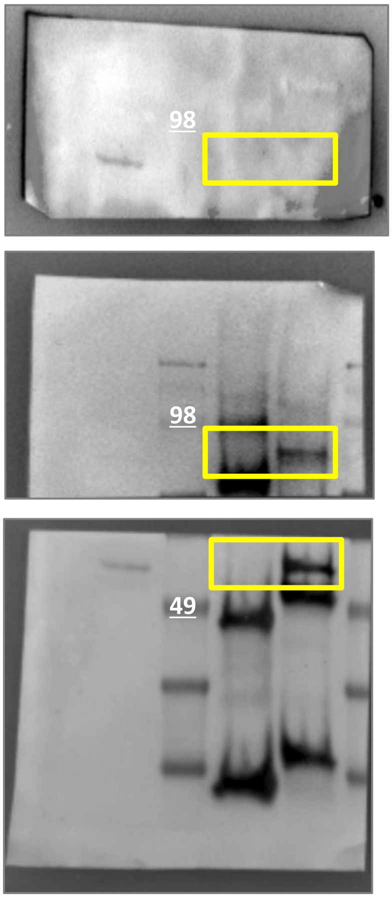

2A (TCE) Kda

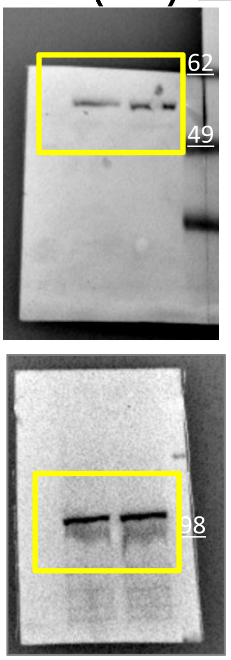

Kda 2A (IP)

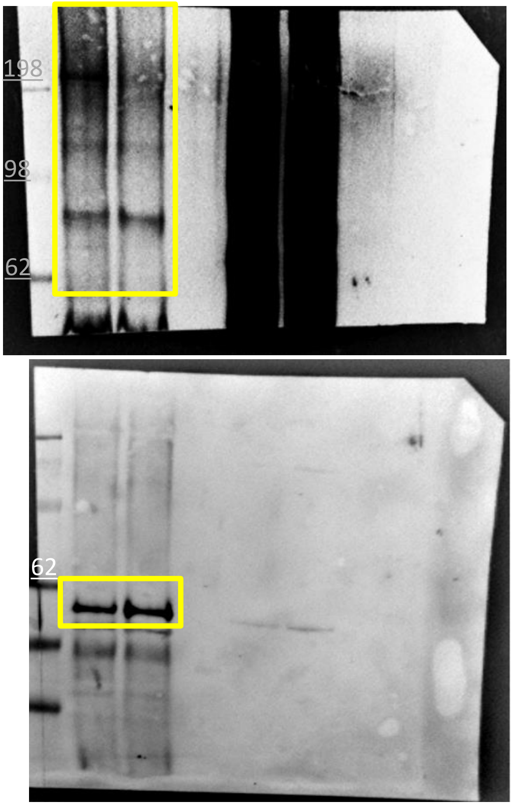

2B Kda

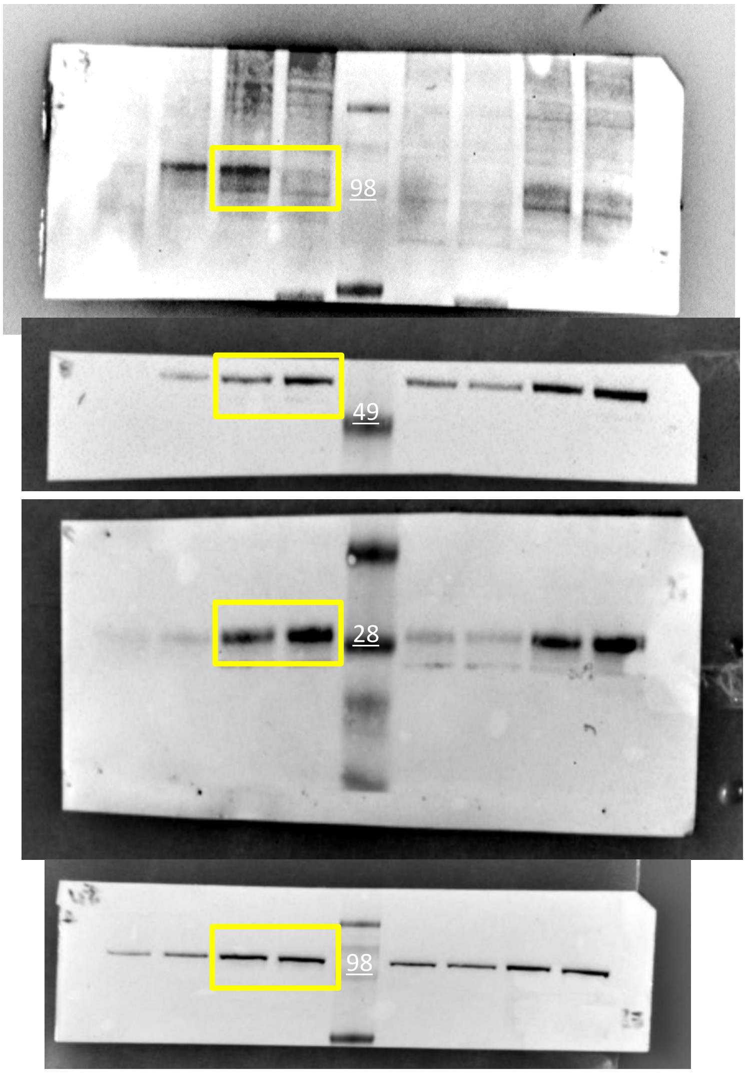

2C Kda

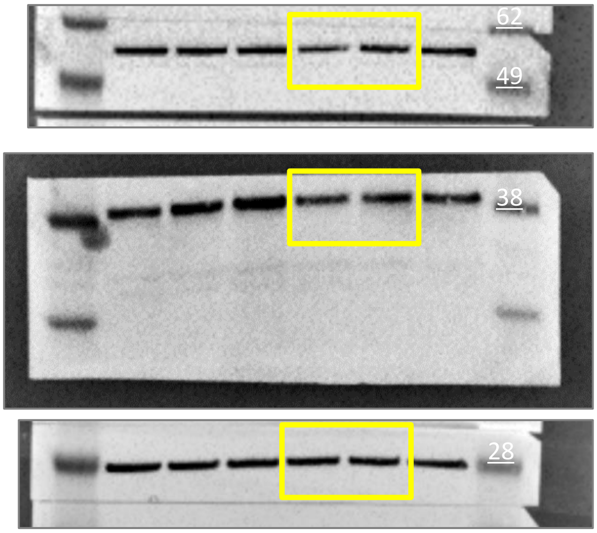

3I

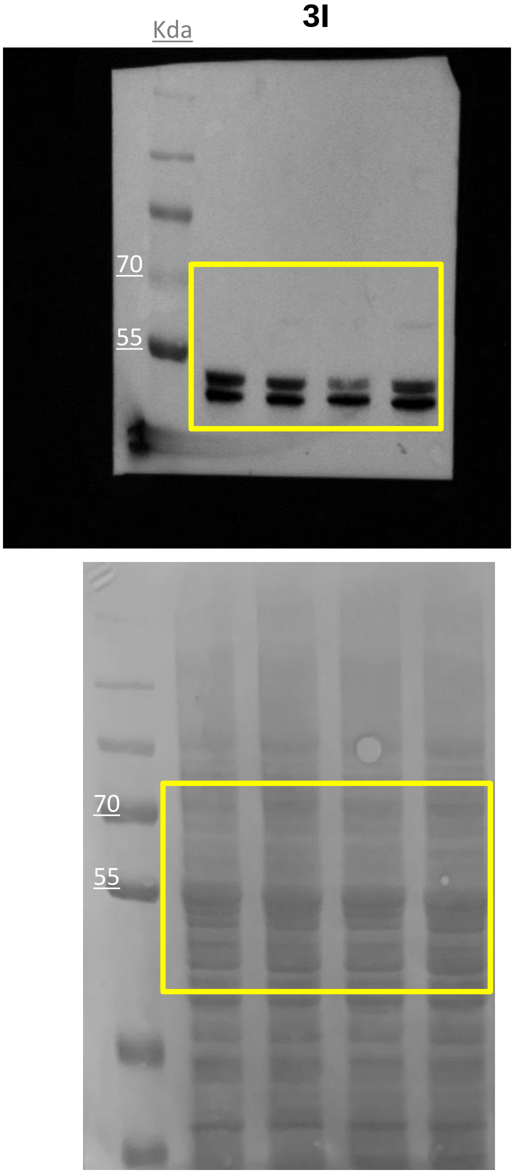

5C

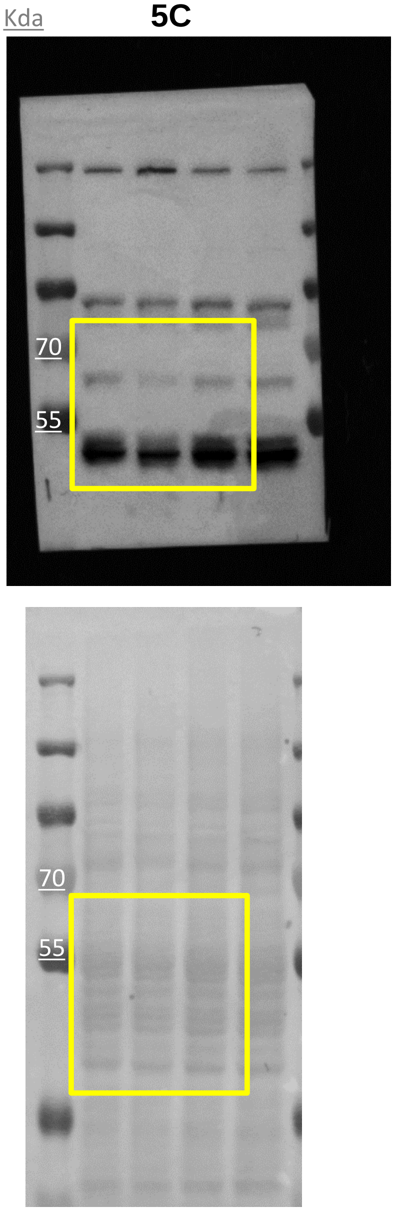

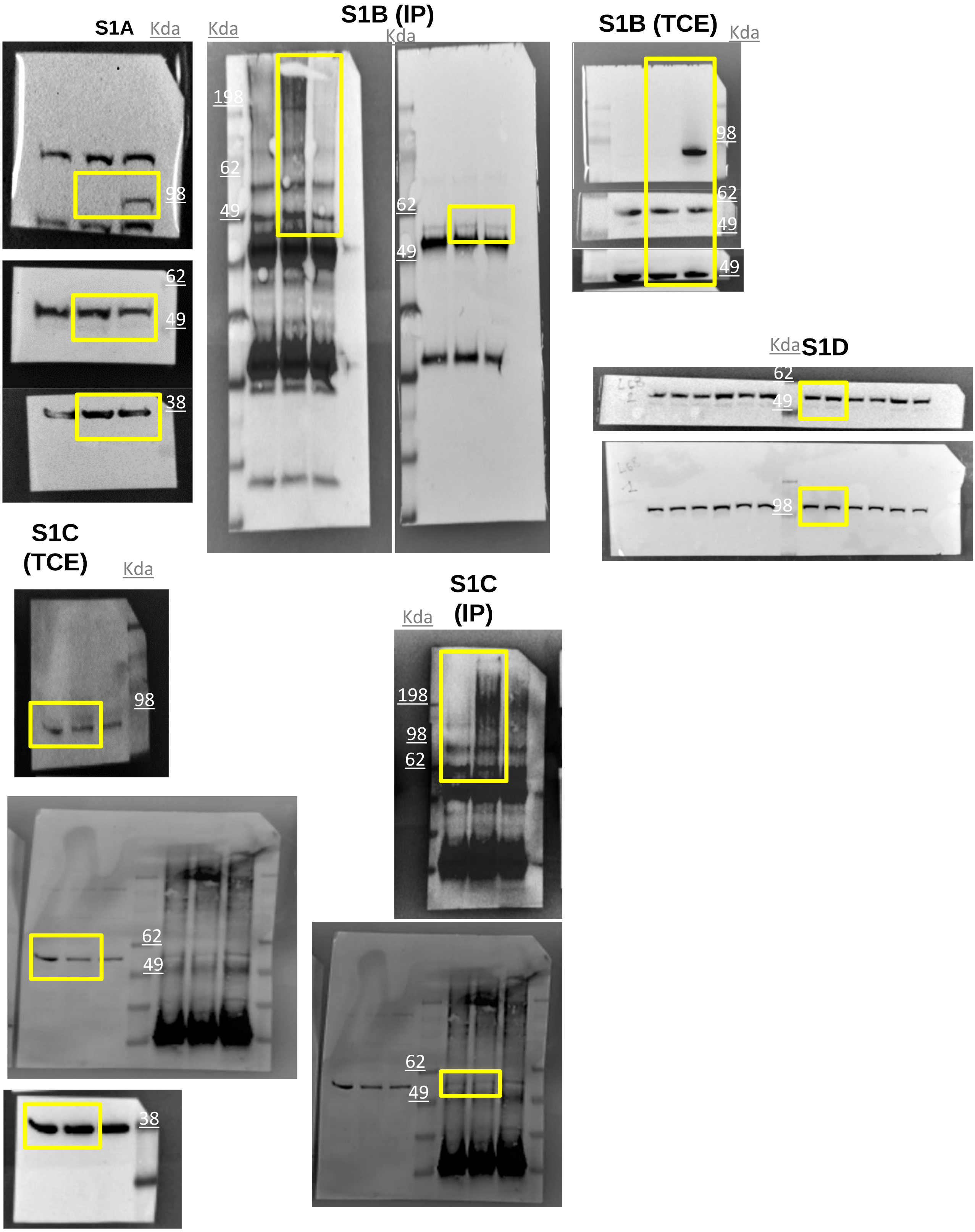
S1A
Kda
98
62
49
38
S1B (IP)
Kda
198
62
49
Kda
62
49
S1B (TCE)
Kda
98
62
49
49
Kda
S1D
62
49
98
S1C
(TCE)
Kda
98
62
49
38
S1C
(IP)
Kda
198
98
62
62
49

S1F

Kda

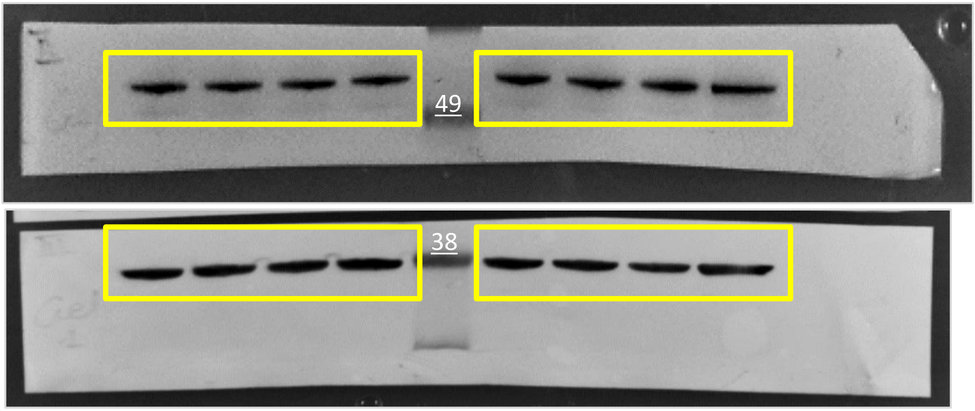

S1E (TCE) Kda **S1E (IP)**

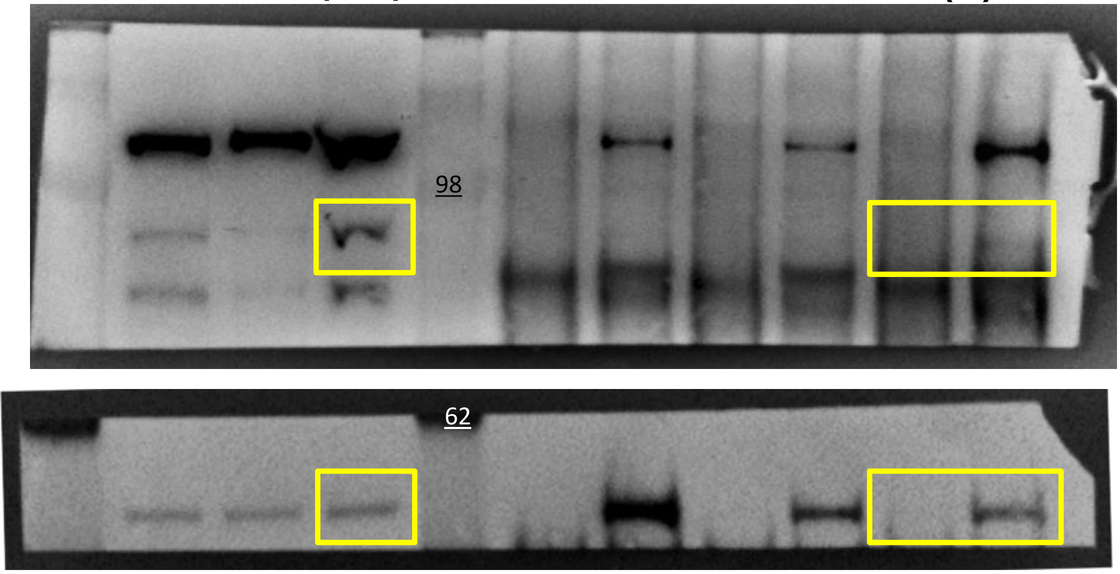

S1F (TCE) Kda **S1F (IP)**

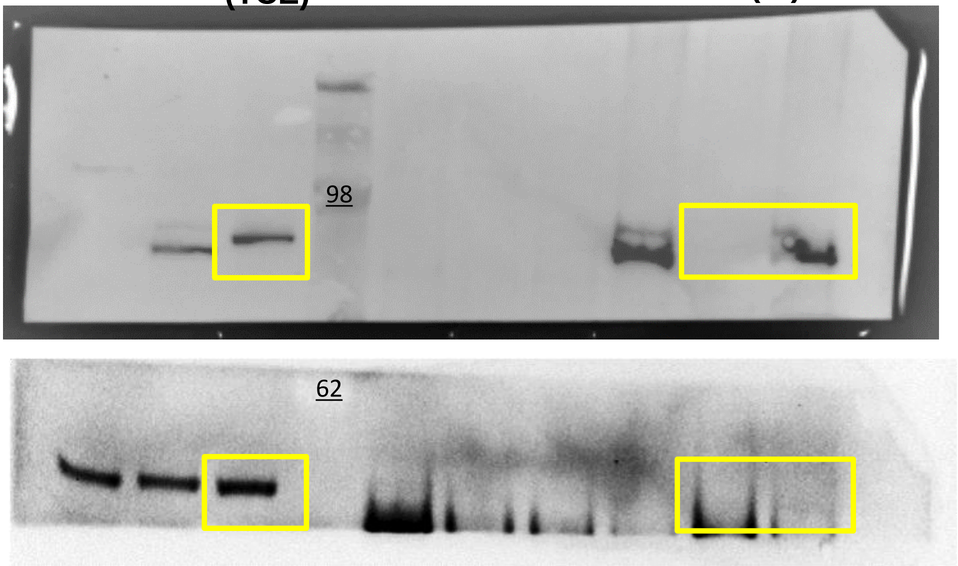

Supplement: awae095_Supplementary_Data [file awae095_supplementary_data.zip › brain-2023-01619-File008.pdf]
